# Exercise reprograms the gut microbiota to enhance metabolic outcomes after bariatric surgery: a translational, cross-species study

**DOI:** 10.1038/s41366-026-02121-3

**Published:** 2026-06-04

**Authors:** Alisson Padilha de Lima, Juliana Bueno de Novais, Adriane Elisabete Costa Antunes, Matteo Calgaro, Carlos Merege-Filho, Diego Nunes Rezende, Marina Alvarenga Romero, Heloisa Castanheira Santo André, Igor Baptista, Ana Lúcia de Sá Pinto, Roberto de Cleva, Marco Aurélio Santo, Wagner Silva Dantas, Saulo Gil, Maria Rita Camargo Rodrigues, Guilherme Gianini Artioli, Nicola Vitulo, Hamilton Roschel, Bruno Gualano, Carolina Ferreira Nicoletti, Fabiana Braga Benatti

**Affiliations:** 1 Applied Physiology and Nutrition Research Group – School of Physical Education and Sport and School of Medicine, University of Sao Paulo, Sao Paulo, SP Brazil; 2 Center of Lifestyle Medicine, School of Medicine, University of Sao Paulo, Sao Paulo, SP Brazil; 3 Laboratory of Assessment and Conditioning in Rheumatology; Hospital das Clínicas HCFMUSP, Faculdade de Medicina FMUSP, Universidade de Sao Paulo, São Paulo, SP Brazil; 4 School of Physical Education, Faculty IELUSC, Joinville, SC Brazil; 5 Universidade Estadual de Campinas, Faculdade de Ciencias Aplicadas, Limeira, SP Brazil; 6 Department of Biotechnology, University of Verona, Verona, Italy; 7 Gastroenterology Department, Digestive Surgery Division, Faculdade de Medicina - FMUSP, Universidade de São Paulo, São Paulo, SP Brazil; 8 Department of Human Physiology, University of Oregon, Eugene, OR USA; 9 Laboratory of Nutritional & Exercise Biology, Department of Anatomy, Institute of Biomedical Sciences, University of Sao Paulo, Sao Paulo, Brazil

**Keywords:** Translational research, Bacteria

## Abstract

**Background/Objectives:**

Exercise training has been associated with metabolic improvements in bariatric patients beyond weight and fat loss, potentially involving modulation of the gut microbiota. We investigated whether exercise-related microbial changes are associated with metabolic adaptations in women undergoing Roux-en-Y gastric bypass (RYGB) by combining a randomized controlled exercise intervention trial in women post-surgery with a human-to-mouse fecal microbiota transplantation (FMT) experiment.

**Subjects/Methods:**

Thirty-two women were randomized to RYGB (*n* = 16) or RYGB plus a 6-month exercise training program initiated three months post-surgery (RYGB + ET; *n* = 16), while a lean control group (LEAN; *n* = 16) was evaluated at baseline. Blood and fecal samples were collected before surgery, and at 3 (POST3) and 9 (POST9) months following surgery for biochemical, inflammatory, and microbiota analyses.

**Results:**

Both surgical groups showed comparable improvements in body composition and inflammation; however, RYGB + ET was associated with greater improvements in HDL, triglycerides, fasting glucose, and fasting insulin. Exercise was also associated with increased gut microbiota α-diversity and shifts in microbial composition, including enrichment of genera previously linked to short-chain fatty acid (SCFA) metabolism and host metabolic health. To explore the potential contribution of these microbial communities, fecal microbiota collected at POST9 were transplanted into 36 high-fat diet-fed female mice, generating recipient groups rRYGB, rRYGB+ET, and rLEAN. Mice receiving RYGB + ET microbiota displayed similar inflammatory status and glucose tolerance, but lower fasting insulin and HOMA-IR, along with partial preservation of intestinal morphology, compared with mice receiving RYGB microbiota.

**Conclusions:**

These findings suggest that exercise following bariatric surgery is associated with distinct gut microbial configurations and metabolic improvements, and that exercise-conditioned microbiota may contribute to aspects of host metabolic regulation after surgery.

## Introduction

The gut microbiota plays a pivotal role in modulating host physiology, metabolism, and immune function, influencing key cardiometabolic risk factors, including insulin sensitivity, systemic inflammation, glucose and lipid metabolism [1–3]. Bariatric surgery is a highly effective intervention for individuals with BMI > 40 Kg/m^2^ or BMI > 35 Kg/m^2^ with obesity-related complications [4, 5], leading to substantial weight loss and improvements in cardiometabolic health [6–10]. Increasing evidence indicates that bariatric surgery also induces significant shifts in gut microbiota composition, often characterized by increased diversity and alterations in specific bacterial taxa, such as *Escherichia*, *Akkermansia*, *Streptococcus*, *Veillonella*, *Gemella*, *Fusobacterium* [11–14]. Importantly, fecal microbiota transplantation (FMT) studies in mice have demonstrated that these microbial changes can transfer metabolic benefits independently of weight loss, highlighting a causal role for the microbiota in mediating surgical outcomes [15–17].

Exercise training is a cornerstone of obesity management and has been shown to confer additive metabolic benefits in bariatric patients, including improved insulin sensitivity [18, 19] reduced inflammation [19], enhanced endothelial function [19], and preservation of muscle and bone mass [20, 21] independently of weight and fat loss. Moreover, exercise has emerged as a potent modulator of gut microbiota composition in individuals with overweight and obesity, with some studies reporting increased microbial diversity and enrichment of genera such as *Bacteroides* and *Akkermansia* [22–26]. Given the established links between gut microbiota and metabolic health, exercise-induced shifts in microbial composition may help to explain the additional metabolic benefits seen in exercised patients following bariatric surgery. However, this hypothesis has not yet been directly tested.

We conducted a translational, cross-species study to explore whether gut microbiota may contribute to the metabolic benefits of exercise after bariatric surgery, by combining a randomized controlled exercise intervention trial in women post-surgery with a human-to-mouse FMT experiment. We aimed to 1) determine how exercise training alters gut microbiota diversity and composition in patients with obesity undergoing bariatric surgery; and 2) evaluate whether these exercise-induced microbial changes are sufficient to reproduce metabolic benefits in diet-induced mice with obesity model.

## Methods

### Study design

This study is part of a randomized controlled clinical trial designed to investigate the benefits of exercise training in women undergoing bariatric surgery [19–21, 27] (NCT02441361), approved by the Research Ethics Committee of the General Hospital of the Faculty of Medicine of the University of São Paulo.

**Fig. 1:**
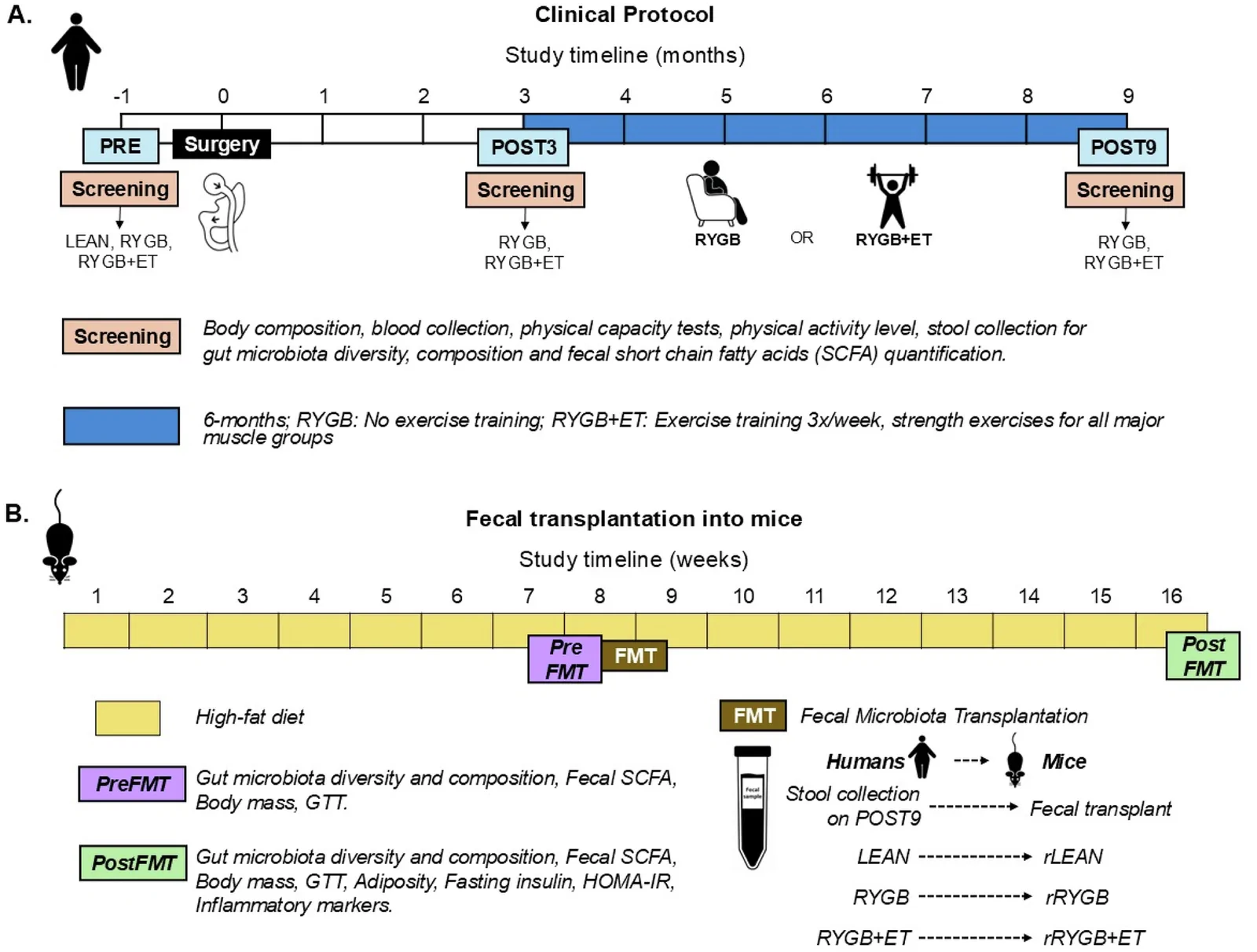
Experimental design. **A** Clinical trial; **B** Fecal microbiota transplant study.

The present dataset focuses on the effects of exercise training on gut microbiota diversity, composition, and related metabolic outcomes after bariatric surgery. Following baseline assessments, 32 women were randomly assigned (1:1), using a computer-generated sequence, to Roux-en-Y gastric bypass alone (RYGB; *n* = 16) or Roux-en-Y gastric bypass plus exercise training (RYGB + ET; *n* = 16). The six-month supervised exercise training sessions for the RYGB + ET has been detailed elsewhere [19–21], happened three times a week and comprised strengthening exercises for the major muscle groups and moderate-intensity aerobic exercise on a treadmill. Adverse effects and attendance were recorded. Assessments were conducted at baseline (PRE), three months after surgery (POST3), and six months after completion of the exercise training program (POST9). Outcomes included gut microbiota diversity and composition, fecal short-chain fatty acids (SCFA; acetate, propionate, and butyrate), anthropometry, body composition (DEXA), biochemical markers (lipid markers, C-peptide, glycated hemoglobin and insulin resistance markers Homeostatic Model Assessment for Insulin Resistance [HOMA-IR], calculated using fasting blood glucose and insulin levels, and the total areas under the curve for insulin [AUC_insulin_] and glucose (AUC_glucose_], calculated from a 3-h oral glucose tolerance test), inflammatory markers (C-reactive protein – CRP, IL-1β, TNF-α, IL-6, and IL-10), physical capacity (VO_2peak_ and lower- and upper-limb maximal dynamic strength) and physical activity level (accelerometry), and food intake (three 24-h dietary recalls). For reference, an age- and sex-matched lean control group (LEAN; *n* = 16) was evaluated at baseline only (Fig. 1A).

We next colonized 36 SPF female mice who had undergone an eight-week high-fat diet with either 1) fecal microbiota of participants of RYGB collected at POST9 (*n* = 12; rRYGB); 2) fecal microbiota of participants of RYGB + ET collected at POST9 (rRYGB+ET; *n* = 12); or 3) fecal microbiota of participants of LEAN (*n* = 12; rLEAN). Colonization was performed by oral gavage for eight weeks while animals remained on the high-fat diet. Before (PREFMT) and after colonization (POSTFMT), body weight, 2-h oral glucose tolerance (following a 4-h fast), gut microbiota composition, and fecal SCFA were assessed. At POSTFMT, adiposity, insulin resistance marker HOMA-IR (calculated using fasting blood glucose and insulin levels), inflammatory cytokines (IL-1β, TNF-α, IL-6, and IL-10), intestinal morphology (crypt depth, villi height and width, villi/crypt ratio and inflammatory histological score of the colon and jejunum) and adipose tissue gene expression (IL-1β, TNF-α, IL-6, and IL-10) were evaluated (Fig. 1B). This study was approved by the Animal Ethics Committee of the Institute of Biomedical Sciences of the University of São Paulo.

Due to space constraints, descriptions of physical capacity and level testing, anthropometry, food intake, body composition, biochemical analyses, systemic inflammation, intestinal morphology, adipose tissue gene expression, and fecal SCFA quantification are provided in Supplementary Information.

### Human participants

Participants were recruited from the Metabolic and Bariatric Surgery Unit of the Clinical Hospital of the School of Medicine at the University of Sao Paulo. Eligible participants were women aged 18–60 years with BMI > 40 kg/m² or ≥35 kg/m² with obesity-related comorbidities and not engaged in structured exercise training for at least one year prior to enrollment. Exclusion criteria included pregnancy, cancer within the previous five years, gastrointestinal disease, use of medications affecting gastrointestinal function, cardiovascular disease, use of antibiotics three months prior to the study, and neurological or musculoskeletal conditions contraindicating exercise. The LEAN group followed the same exclusion criteria and comprised physically active women (i.e., achieving a minimum of 21.4 min/day of moderate-to-vigorous physical activity) [28] with BMI between 18.5 and 24.9 kg/m² [27].

Data were collected between February 2019 and July 2023 in Sao Paulo (Brazil). All procedures complied with the Declaration of Helsinki, and all patients provided written informed consent.

### Fecal collection

Participants were instructed to avoid probiotic-containing foods for seven days prior to collection. On the eighth day, the first stool of the day was collected using two sterile Falcon tubes: one containing 5 mL of 6 M guanidine solution for microbiota analysis and one empty tube for SCFA analysis and fecal microbiota transplantation. Samples were stored at −80 °C until analysis. In the RYGB + ET group, stool was collected 48–72 h after the last training session. While stool consistency was not formally graded using the Bristol Stool Chart, all samples were collected and processed using a standardized protocol to ensure consistency in microbial and SCFA analysis.

### Fecal microbiota transplant (FMT)

Fecal samples collected at POST9 from RYGB, RYGB + ET, and LEAN participants (~500 mg) were pooled, homogenized in sterile saline with 10% glycerol, filtered, and stored at −80 °C [29]. Each pool consisted of samples from five randomly selected participants per group and was analyzed for microbiota diversity and composition to infer engraftment in the recipient mice groups. To reduce endogenous microbiota, mice received antibiotics gentamicin, ampicillin, metronidazole, neomycin, and vancomycin (Sigma®) in drinking water for three days prior to transplantation. From day 4 onward, animals received oral gavage of 100 mg fecal slurry twice-a-week for eight weeks.

### Animals

Female SPF C57BL6/N mice (4–5 weeks old) were housed in microisolator cages (four per cage) under controlled conditions, with ad libitum access to food and water at the Central Animal Facility of the School of Medicine of the University of Sao Paulo, SP, Brazil. Animals were fed a high-fat diet (Rhoster® 45% fat) throughout the experiment. Fecal microbiota transplantation was initiated after eight weeks on the diet.

### Euthanasia and collection of biological material

At POSTFMT, mice were fasted for 12 h and euthanized by intraperitoneal injection of xylazine and ketamine. Blood was collected for biochemical and inflammatory analyses. Adipose tissue depots were excised and weighed to determine adiposity. Visceral adipose tissue and gastrocnemius muscle were stored at −80 °C for molecular analyses. Intestinal samples were fixed in methanol:chloroform:acetic acid (6:3:1) and stored in 70% ethanol at 4 °C for histological analysis.

### Gut microbiota sequencing and bioinformatics analysis

Fecal DNA extraction was performed using the MagMax™ CORE Nucleic Acid Purification Kit (Thermofisher). DNA quality and quantity were assessed by agarose gel electrophoresis and Nanodrop®. Bacterial community profiling was performed by high-throughput sequencing of the V3–V4 regions of the 16S rRNA gene. Library preparation was performed according to the protocol of Neoprospecta Microbiome Technologies (Brazil). Libraries were sequenced on an Illumina MiSeq platform (2× 300 bp, MiSeq Reagent Kit v3).

Sequencing data were processed in R (v4.4.2) using DADA2. After primer removal, reads were quality filtered and truncated (human: 282/179 bp; mouse: 283/173 bp). Amplicon sequence variants (ASVs) were inferred, chimeras were removed using DADA2’s consensus method, and taxonomy was assigned using the RDP database (v19, 2024 release). ASVs were subsequently agglomerated at the genus level using the mia package (v1.14.0).

Microbial community structure was characterized in terms of α- and β-diversity. α-diversity (richness, Shannon index, dominance) and β-diversity were calculated, with community structure visualized using principal coordinates analysis of Aitchison distances.

### Sample size and statistical treatment

In the clinical trial, the sample size was determined by feasibility criteria, in accordance with previous recommendations [30, 31], considering clinic volume, surgical capacity, and staff availability, resulting in 32 participants, consistent with prior studies [25]. For the animal study, we estimated a sample size of 12 mice per group based on previous studies using human-to-mouse FMT [32], considering that this number provides sufficient statistical power to detect significant shifts in microbial composition and host metabolic characteristics, in addition to possible losses that can vary from 5 to 10% of the fecal sample.

A linear mixed model for repeated measures analysis was used, with group, time, and the group-by-time interaction considered as fixed factors, and subjects as random factors. Residual analyses were used to determine the presence of extreme observations and the normal distribution of residuals. Tukey post hoc was used for multiple comparisons. All analyses were adjusted for PRE values. Delta scores (POST9 – PRE) were also calculated for the primary dependent variables and analyzed using a mixed model in the clinical study, adjusted for PRE values. Baseline comparisons used Fisher’s exact tests and unpaired Student’s *t* tests. All analyses followed the intention-to-treat principle. Data are expressed as mean (standard deviation), difference between groups, and 95% CI unless otherwise stated. The significance level adopted to reject the null hypothesis was *p* ≤ 0.050. Results with p-values between 0.05 and 0.10 were considered indicative of a trend and interpreted cautiously as exploratory findings. Data analysis was performed using SAS® OnDemand for Academics (SAS Institute Inc., Cary, NC).

All statistical analyses related to the gut microbiota metabarcoding data were performed in R (version 4.4.2). Global differences in community composition were assessed by permutational analysis of variance (PERMANOVA), with additional tests for homogeneity of within-group dispersions. PERMANOVA analyses were performed using the adonis2 function of the vegan package (version 2.6-10) (i) at baseline (PRE), comparing all groups (LEAN, RYGB, and RYGB + ET), and (ii) across all time points (PRE, POST3, and POST9), restricted to the RYGB and RYGB + ET groups, since the LEAN group was sampled once.

Temporal effects within and between groups were assessed using generalized linear mixed-effects models fitted with the lmerTest (v3.1-3) package for α-diversity and glmmTMB (version 1.1.11) package for genus-level differential abundance analysis. α-diversity metrics and genus-level centered log ratio (CLR) values were used as response variables, with group, time, and the group × time interaction considered as fixed factors. Models included random intercepts for patient or mouse identifiers, and Sidak-adjusted pairwise contrasts were applied.

In addition to statistical significance (*p* < 0.05), biological relevance thresholds were applied for α-diversity (absolute differences >10% of PRE values). Microbial abundance results were filtered using false discovery rate (FDR) correction (*p* < 0.05).

## Results

### Participants

A total of 302 women were screened, 32 met the inclusion criteria and were randomized (1:1) to the Roux-en-Y Gastric Bypass (RYGB; *n* = 16) or RYGB plus exercise training group (RYGB + ET; *n* = 16). Sixteen lean women (LEAN) were assessed at baseline (Supplementary Fig. 1). All participants completed the intervention; however, at POST3 and POST9, 6 and 2 patients from the RYGB group, respectively, provided only fasting blood samples. Exercise attendance was 81.5 ± 13.1%, and no adverse events occurred. Groups were comparable at baseline, except for the prevalence of anxiolytic use, and triglyceride levels, which were higher in RYGB + ET (Table 1). Medication reductions occurred similarly in both groups and no antibiotics were used during the trial (Supplementary Table 1).

**Table 1:** Characteristics of patients and healthy controls at baseline.

Variables	RYGB (*n* = 16)	RYGB + ET (*n* = 16)	*P* value	LEAN (*n* = 16)
Age (years)	39.3 ± 8.1	44.3 ± 9.2	0.11	38.1 ± 2.2
Coronary artery disease	0 (0.0)	0 (0.0)	1	0 (0.0)
Hypertension	8 (50.0)	11 (68.8)	0.47	0 (0.0)
Type 2 diabetes	7 (43.8)	11 (68.8)	0.14	0 (0.0)
Alcoholism	6 (37.5)	4 (25.0)	0.70	0 (0.0)
Hypothyroidism	3 (18.7)	4 (25.0)	0.69	0 (0.0)
Smoking	0 (0.0)	0 (0.0)	1	0 (0.0)
Ethnicity				
Black	3 (18.8)	3 (18.8)	1	0 (0.0)
White	10 (62.5)	6 (37.5)	0.29	11 (68.8)
Brown	3 (18.8)	6 (37.5)	0.43	5 (31.2)
Indigenous	0 (0.0)	1 (6.3)	1	0 (0.0)
Education				
Elementary	1 (6.3)	1 (6.3)	1	0 (0.0)
Secondary	13 (81.3)	11 (68.8)	0.68	4 (25.0)
Higher education	2 (12.5)	4 (25.0)	0.65	12 (75.0)
Socioeconomic (ABEP)				
High	0 (0.0)	1 (6.3)	1	1 (6.3)
Medium	6 (37.5)	5 (31.3)	1	9 (56.3)
Low	10 (62.5)	10 (62.5)	1	6 (37.5)
Medications				
Antibiotics	0 (0.0)	0 (0.0)	1	0 (0.0)
Anxiolytics	0 (0.0)	4 (25.0)	0.05	0 (0.0)
Antiepileptic	2 (12.5)	0 (0.0)	0.24	0 (0.0)
Alpha-2 adrenergic agonist	2 (12.5)	0 (0.0)	0.24	0 (0.0)
A-II receptor antagonist	1 (6.3)	2 (12.5)	1	0 (0.0)
Calcium blocker	0 (0.0)	2 (12.5)	0.24	0 (0.0)
Diuretic	4 (25.0)	7 (43.8)	0.46	0 (0.0)
ACE inhibitors	0 (0.0)	1 (6.3)	1	0 (0.0)
Insulin	0 (0.0)	0 (0.0)	1	0 (0.0)
Metformin	7 (43.8)	11 (68.8)	0.28	0 (0.0)
Anthropometric measurements and body composition				
Body weight (kg)	123.18 ± 21.44	117.48 ± 20.17	0.43	61.59 ± 8.49
BMI (kg/m^2^)	38.61 ± 6.45	37.43 ± 6.46	0.61	19.02 ± 3.04
WC (cm)	119.23 ± 11.45	122.83 ± 11.96	0.76	73.34 ± 9.62
Fat percentage (%)	51.36 ± 4.40	52.73 ± 3.82	0.36	30.86 ± 1.59
Biochemical parameters				
HDL cholesterol (mg/dL)	51.19 ± 9.63	45.44 ± 7.79	0.12	71.44 ± 22.95
LDL cholesterol (mg/dL)	114.56 ± 32.63	120.69 ± 29.46	0.58	105.00 ± 28.89
Triglycerides (mg/dL)	113.44 ± 40.41	152.00 ± 62.57	0.01	87.75 ± 41.78
Total cholesterol (mg/dL)	183.00 ± 32.17	196.56 ± 35.06	0.26	157.63 ± 27.85
C-reactive protein(mg/dL)	11.38 ± 9.48	14.33 ± 10.18	0.40	1.63 ± 1.01
Fasting glucose (mg/dL)	123.50 ± 59.33	122.75 ± 52.38	0.33	84.63 ± 7.67
Fasting insulin (μIU/mL)	26.20 ± 15.32	30.53 ± 13.62	0.19	7.44 ± 4.68
HOMA-IR	8.41 ± 8.20	9.69 ± 6.44	0.63	1.57 ± 1.03
Physical capacity and activity level				
VO2peak (ml/Kg/min)	15.52 ± 3.38	14.04 ± 2.85	0.92	35.05 ± 8.10
1-RM leg-press (Kg)	210.00 ± 56.24	191.25 ± 53.81	0.92	180.63 ± 62.66
1-RM bench-press (Kg)	38.56 ± 8.60	37.60 ± 9.88	0.99	27.69 ± 6.87
Sedentary time (min/day)	558.78 ± 210.51	593.61 ± 85.34	0.58	454.33 ± 155.72
LPA (min/day)	79.71 ± 34.45	78.09 ± 24.27	0.89	88.71 ± 25.11
MVPA (min/day)	6.82 ± 9.99	9.80 ± 10.48	0.46	42.48 ± 22.37

Data expressed as mean ± standard deviation or n (%).

*ABEP* Brazilian Association of Research Companies, *A-II* Angiotensin-II, *ECA* Angiotensin Converting Enzyme, *BMI* Body Mass Index, *WC* Waist circumference, *LPA* low-intensity physical activity, *MVPA* moderate-to-vigorous-intensity physical activity.

### Exercise training preserved lean mass and enhanced the metabolic profile after RYGB surgery

Both RYGB and RYGB + ET groups exhibited substantial reductions in body weight (33.7% and 32.0%, respectively), waist circumference, visceral fat, and fat mass across time, with no between-group differences. However, the RYGB + ET group showed a significantly attenuated decrease in lean mass and greater improvements in moderate-to-vigorous physical activity level (MVPA), cardiorespiratory fitness (VO₂_peak_), lower-body strength (1-RM leg press), and upper-body strength (1-RM bench press) compared with the RYGB group, confirming the efficacy of the exercise training intervention (Supplementary Table 2).

Both the RYGB and RYGB + ET groups showed reductions in all dietary intake parameters (total energy, carbohydrates, protein, lipids, and fiber) across time, with no significant differences between groups (Supplementary Table 2).

**Fig. 2:**
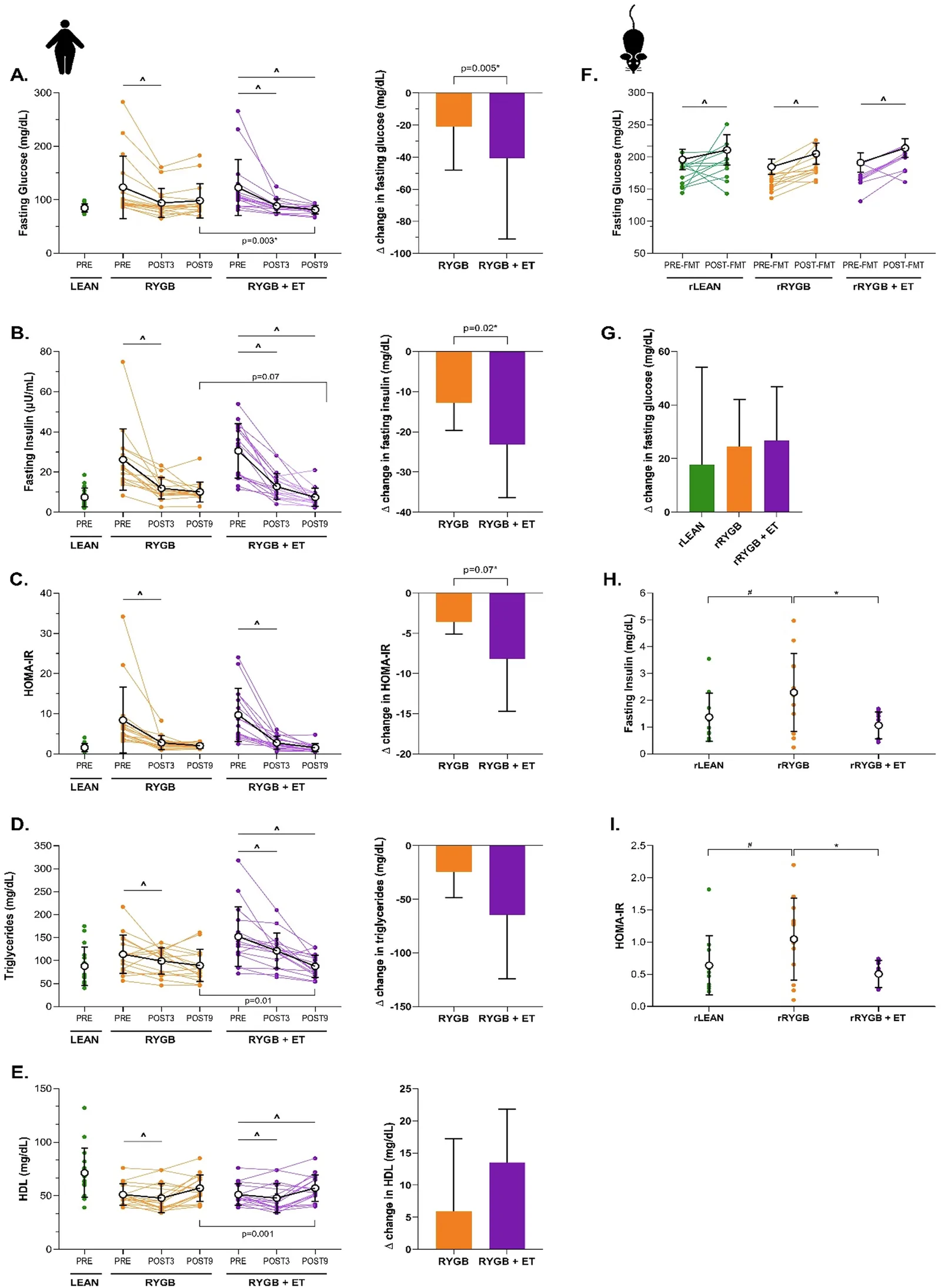
Fasting insulin sensitivity indexes in trained and non-trained bariatric women and lean controls, and in SPF female mice with obesity who received their fecal microbiota transplant (FMT). **A** Change in fasting glucose after surgery in trained and non-trained bariatric women and lean controls. **B** Change in fasting insulin after surgery in trained and non-trained bariatric women and lean controls. **C** Change in HOMA-IR after surgery in trained and non-trained bariatric women and lean controls. **D** Change in triglycerides after surgery in trained and non-trained bariatric women and lean controls. **E** Change in HDL-cholesterol after surgery in trained and non-trained bariatric women and lean controls. **F** and **G** Change in fasting glucose in mice receiving FMT of trained and non-trained bariatric women and lean controls. **H** Change in fasting insulin in mice receiving FMT of trained and non-trained bariatric women and lean controls. **I** Change in HOMA-IR in mice receiving FMT of trained and non-trained bariatric women and lean controls. Data are presented as mean ± standard deviation and Delta (∆) change = POST9 – PRE for A, B and C; POSTFMT – PREFMT for (**E**). ^ indicates significant main effects of time; * indicates significant between-group differences.

C-reactive protein (CRP) levels decreased similarly in both groups, but other systemic inflammatory markers remained unchanged. Despite comparable effects on inflammation, RYGB + ET showed greater improvements in lipid and glycemic profiles, including higher HDL, lower triglyceride, lower fasting glucose and c-peptide and non-significant tendency toward lower fasting insulin levels (*p* = 0.07) at POST9 compared with RYGB (Table 2 and Fig. 2A–E). These findings indicate that exercise augments the metabolic benefits of surgery that are not explained by changes in body composition, food intake or systemic inflammation.

**Table 2:** Effects of exercise training on metabolic and inflammatory parameters in women undergoing bariatric surgery.

Variables	RYGB (*n* = 16)	RYGB + ET (*n* = 16)	Difference (IC - 95%)	*P*-value	*P*-value Group x Time
Total cholesterol (mg/dL)					0.253
PRE^a^	183.00 ± 33.22	196.56 ± 36.20	−3.94 (−20.62 to 12.73)	0.63	
POST3^b^	155.40 ± 33.77	172.81 ± 39.94	−6.24 (−23.21 to 10.72)	0.46	
POST9^b^	162.50 ± 33.30	162.44 ± 25.21	9.68 (−6.99 to 26.36)	0.25	
∆ change	−20.50 ± 24.55	−34.12 ± 30.02	7.52 (−9.38 to 24.43)	0.37	
LDL cholesterol (mg/dL)					0.580
PRE^a^	114.56 ± 33.70	120.69 ± 30.42	−1.58 (−15.90 to 12.72)	0.82	
POST3^b^	90.13 ± 34.23	102.94 ± 31.10	−6.42 (−20.96 to 8.12)	0.38	
POST9^c^	87.00 ± 32.79	88.81 ± 23.23	2.72 (−11.59 to 17.03)	0.70	
∆ change	−27.56 ± 24.87	−31.87 ± 24.83	1.81 (−13.79 to 17.42)	0.81	
HDL cholesterol (mg/dL)					0.044
PRE	51.19 ± 9.95	45.44 ± 8.04	5.70 (−4.28 to 5.69)	0.77	
POST3	47.87 ± 13.52	43.75 ± 8.61	−0.51 (−5.56 to 4.53)	0.84	
POST9	57.13 ± 12.46^$^	58.94 ± 9.56*^$^	−6.85 (−11.84 to −1.87)	0.001	
∆ change	5.93 ± 11.28	13.50 ± 8.33	−5.56 (−12.87 to 1.71)	0.12	
Triglycerides (mg/dL)					0.016
PRE	113.44 ± 41.74	152.00 ± 64.63	−16.38 (−35.29 to 2.52)	0.08	
POST3	99.00 ± 28.98*	121.13 ± 38.31*	−0.28 (−19.47 to 18.91)	0.97	
POST9	89.19 ± 34.79*	87.25 ± 24.35*^$^	24.11 (5.20 to 43.02)	0.01	
∆ change	−24.25 ± 31.59	−64.75 ± 59.46	12.32 (−8.20 to 32.85)	0.22	
Fasting glucose (mg/dL)					0.365
PRE^a^	123.50 ± 58.32	122.75 ± 52.38	0.34 (−14.37 to 15.05)	0.96	
POST3^b^	94.06 ± 26.86	88.68 ± 13.06	4.96 (−9.75 to 19.68)	0.50	
POST9^b^	98.43 ± 31.91	81.43 ± 7.78	16.59 (1.87 to 31.30)	0.03	
∆ change	−21.00 ± 26.98	−40.68 ± 50.39	17.04 (5.50 to 29.30)	0.005	
Fasting insulin (μU/mL)					0.21
PRE^a^	26.20 ± 15.31	30.53 ± 16.62	−2.43 (−7.46 to 2.59)	0.33	
POST3^b^	11.90 ± 5.37	12.79 ± 6.35	0.30 (−4.78 to 5.39)	0.90	
POST9^c^	10.11 ± 5.00	7.41 ± 4.58	4.59 (−0.43 to 12.02)	0.07	
∆ change	−12.82 ± 6.81	−23.21 ± 13.15	4.04 (0.46 to 7.62)	0.02	
Fasting C-peptide (ng/mL)					0.074
PRE	3.37 ± 0.96	3.74 ± 0.87	−0.15 (−0.54 to 0.23)	0.43	
POST3	2.27 ± 0.70*	2.51 ± 0.82*	−0.03 (−0.43 to 0.36)	0.87	
POST9	1.92 ± 0.67*	1.70 ± 0.53*	0.43 (0.03 to 0.82)	0.03	
∆ change	−1.45 ± 1.10	−2.04 ± 0.64	0.29 (−0.07 to 1.23)	0.07	
HOMA-IR					0.714
PRE^a^	8.40 ± 8.19	9.68 ± 6.65	−0.79 (−3.24 to 1.65)	0.51	
POST3^b^	2.76 ± 1.74	2.72 ± 1.60	0.51 (−1.93 to 2.95)	0.67	
POST9^b^	2.01 ± 0.60	1.51 ± 1.03	0.82 (−1.63 to 3.28)	0.50	
∆ change	−3.60 ± 1.53	−8.16 ± 6.54	0.62 (−0.07 to 1.31)	0.07	
AUC_glucose_ (mg/dL x h)					0.901
PRE^a^	325.31 ± 165.51	342.76 ± 185.70	−5.43 (−80.41 to 69.54)	0.99	
POST3^b^	261.50 ± 133.30	253.01 ± 105.56	6.36 (−74.74 to 87.46)	0.99	
POST9^b^	231.95 ± 163.68	196.20 ± 52.35	9.65 (−76.11 to 95.43)	0.99	
∆ change	−121.00 ± 105.59	−120.83 ± 133.28	−18.47 (−54.00 to 17.06)	0.29	
AUC_insulin_ (μU/mL x h)					0.817
PRE^a^	158.83 ± 68.90	166.94 ± 74.58	−3.34 (−55.76 to 49.07)	0.99	
POST3^b^	107.53 ± 41.96	140.02 ± 90.35	−20.11 (−77.10 to 36.87)	0.90	
POST9^c^	74.88 ± 21.22	95.12 ± 53.18	−11.22 (−71.39 to 48.95)	0.99	
∆ change	−79.41 ± 71.13	−73.89 ± 84.56	18.66 (−48.45 to 85.78)	0.57	
C-Reactive Protein (mg/dL)					0.159
PRE^a^	11.38 ± 9.79	14.33 ± 10.51	−1.10 (−7.09 to 4.89)	0.99	
POST3^b^	10.48 ± 11.90	7.29 ± 6.22	4.77 (−1.31 to 10.86)	0.20	
POST9^c^	3.62 ± 6.71	2.44 ± 2.87	2.24 (−3.95 to 8.44)	0.89	
∆ change	−8.72 ± 5.53	−11.89 ± 8.62	0.31 (−65.90 to 66.52)	0.99	
TNF (mg/dL)					0.310
PRE	9.27 ± 2.46	10.90 ± 6.72	−0.98 (−3.83 to 1.86)	0.49	
POST3	12.10 ± 6.74	11.22 ± 4.12	1.73 (−1.57 to 5.04)	0.30	
POST9	9.63 ± 3.26	8.20 ± 2.69	2.03 (−0.91 to 4.98)	0.17	
∆ change	0.26 ± 4.56	−2.69 ± 7.17	2.96 (−1.61 to 7.53)	0.19	
IL-6 (mg/dL)					0.225
PRE	34.76 ± 55.7	32.89 ± 44.83	−0.37 (−45.69 to 30.97)	0.98	
POST3	81.50 ± 87.68	37.55 ± 64.00	40.45 (−9.97 to 90.78)	0.18	
POST9	38.50 ± 60.53	27.51 ± 44.91	8.00 (−38.96 to 54.96)	0.99	
∆ change	1.82 ± 75.21	−5.37 ± 51.69	7.20 (−40.55 to 54.95)	0.75	
IL-10 (mg/dL)					0.968
PRE	51.75 ± 82.88	42.16 ± 60.34	2.05 (−35.32 to 39.42)	0.91	
POST3	72.25 ± 113.85	49.16 ± 81.97	9.43 (−34.76 to 53.62)	0.67	
POST9	46.25 ± 76.95	33.00 ± 55.45	3.05 (−65.66 to 41.77)	0.87	
∆ change	−8.84 ± 99.62	−9.15 ± 77.22	−0.30 (−66.62 to 65.09)	0.99	
IL1-ß (mg/dL)					0.332
PRE	4.18 ± 3.42	3.89 ± 3.71	0.12 (−2.64 to 2.89)	0.99	
POST3	3.15 ± 2.23	3.55 ± 2.83	0.45 (−2.99 to 3.89)	0.69	
POST9	5.02 ± 5.05	2.57 ± 1.37	2.16 (−0.70 to 5.03)	0.24	
∆ change	0.69 ± 5.39	−1.31 ± 2.74	2.00 (−1.13 to 5.14)	0.20	

Data as mean ± standard deviation, estimated mean of differences between RYGB + ET and RYGB (95% CI), level of significance (*p*-value; RYGB + ET x RYGB), and level of significance (interaction effect) calculated using a mixed model for repeated measures. Delta (∆) change = POST9 – PRE. Different superscript letters denote significantly different values for the main effect of time (*p* ≤ 0.05). *AUC* area under the curve calculated from the OGTT.

*Means *p* ≤ 0.05 for within-group comparisons (vs. PRE); ^$^Means *p* ≤ 0.05 for within-group comparisons (vs. POST3); ^&^Means *p* ≤ 0.1 for within-group comparisons (vs. PRE).

### Exercise increased gut microbial diversity and enriched SCFA-producing taxa in humans

At baseline, α-diversity did not differ among groups. Following surgery, microbial diversity evaluated by the α-diversity increased in both bariatric groups, but the change reached statistical significance only in the RYGB + ET group (Supplementary Fig. 2).

**Fig. 3:**
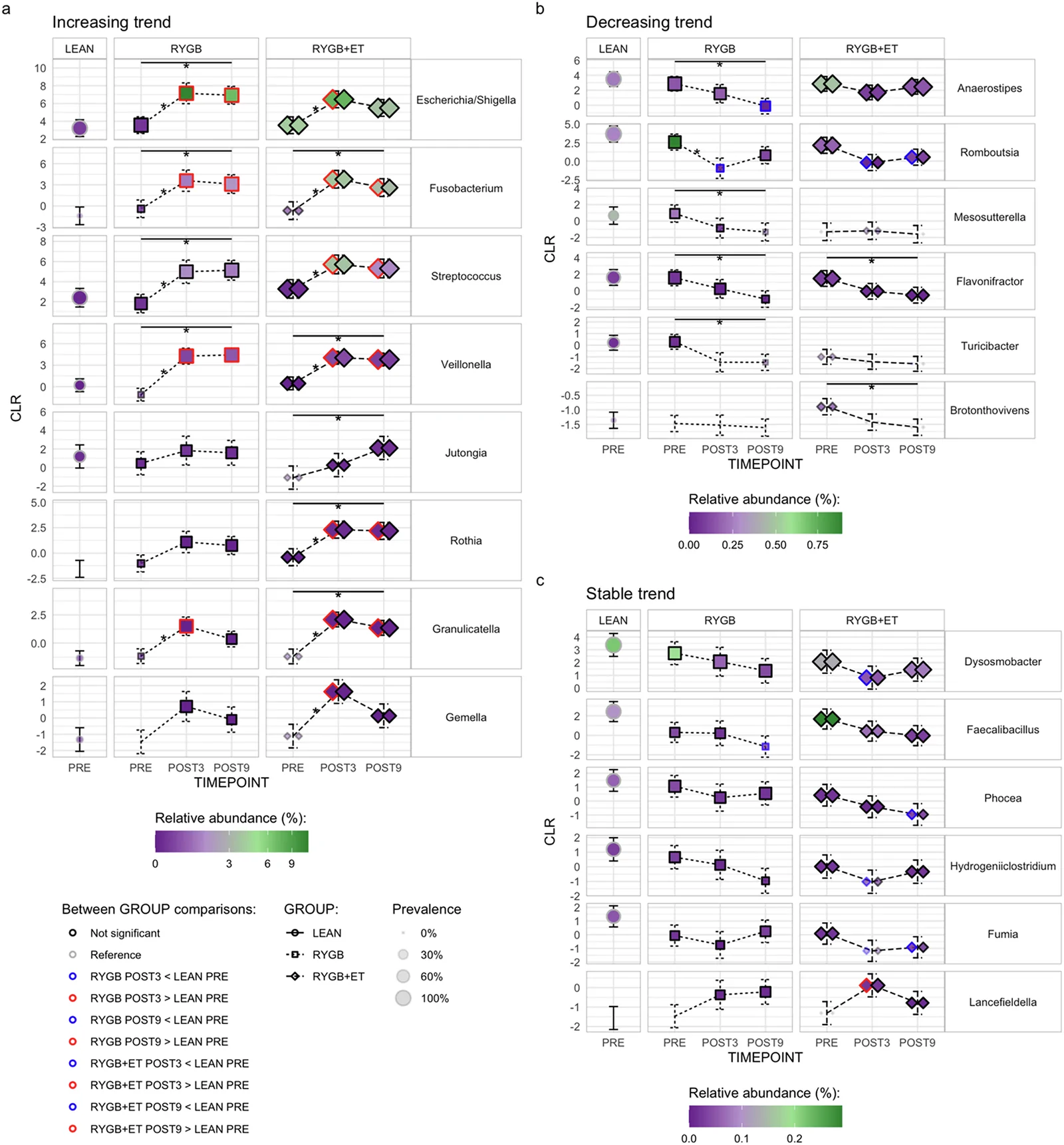
Average centered-log-ratio values (CLR ± 95% confidence interval) for differentially abundant (DA) genera between and/or within GROUP at PRE, POST3, and POST9 after surgery. Circles, squares, and diamonds represent model-estimated CLR values for the LEAN, RYGB, and RYGB + ET groups, respectively, at each time point. Asterisks indicate significant changes across time points within each treatment group. Outline colors indicate the results of between-group contrasts: gray denotes the reference group (LEAN PRE), black a non-significant difference from the reference, blue a significantly lower CLR value than the reference, and red a significantly higher CLR value than the reference. For the RYGB + ET group, two diamonds are shown at each time point: the first refers to the contrast between RYGB + ET and LEAN PRE, and the second to the contrast between RYGB + ET and RYGB at the same time point. Genera in panel (**a**) showed significantly lower CLR values at PRE compared with POST3 or POST9 in at least one group (“increasing” trend). Genera in panel (**b**) showed significantly higher CLR values at PRE compared with POST3 or POST9 (“decreasing” trend). Genera in panel (**c**) showed no significant temporal changes within groups but at least one significant difference between groups (“stable” pattern).

β-diversity analyses showed similar overall shifts in microbial community composition over time in both bariatric groups (Supplementary Fig. 3). However, within-group differential abundance analysis across time revealed that although both RYGB and RYGB + ET experienced changes in genera linked to energy substrate utilization and fermentation, the patterns and direction of these changes differed between groups. Both groups showed increases in genera associated with simple carbohydrate, lactate, and amino acid fermentation (e.g., *Veillonella*, *Granulicatell*a, *Streptococcus*, *Gemella*, *Fusobacterium*), as well as facultative anaerobes such as *Escherichia*/Shigella [11–14]. Nonetheless, exercise training altered this trajectory in the RYGB + ET group by attenuating the loss of short-chain fatty acid (SCFA-producing genera (*Anaerostipes*, *Flavonifractor*, and *Turicibacter)*, limiting the expansion of potential pathobionts such as *Escherichia/Shigella* and *Streptococcus*, and enhancing the relative abundance of *Rothia*, *Jutongia* and Granulicatella, taxa associated with nitrate-reducing pathways that may influence insulin sensitivity through nitric oxide (NO) signaling (Fig. 3a–c) [33].

Fecal SCFAs analysis revealed that acetate decreased at POST3 and returned to baseline at POST9 in both bariatric groups, with no significant differences between RYGB and RYGB + ET. No within- or between-group changes were observed in butyrate or propionate. Therefore, although exercise training altered gut microbiota composition and preserved SCFA-associated taxa, these microbial shifts did not result in detectable changes in fecal SCFA concentrations (Supplementary Table 3).

### Exercise-trained donor microbiota conferred improved insulin sensitivity and intestinal integrity in mice with obesity

To explore the potential contribution of these microbial communities, fecal samples collected at POST9 from LEAN, RYGB, and RYGB + ET participants were transplanted into diet-induced mice with obesity, generating rLEAN, rRYGB, rRYGB+ET groups. We found a 57.4% taxonomic overlap at the genus level between the human donor and the recipient mice (Supplementary Fig. 4). These genera represented 58.4% of the POSTFMT mouse community, aligning with established findings in SPF models [34].

After eight weeks, rRYGB+ET mice exhibited lower fasting insulin and HOMA-IR relative to rRYGB, indicating improved hepatic insulin sensitivity, without changes in glucose tolerance across groups (Fig. 2F–H and Table 3). Body weight, adiposity, circulating inflammatory cytokines (Table 3), and adipose tissue cytokine gene expression (Supplementary Fig. 5) also did not differ between groups, suggesting that the metabolic improvement was independent of systemic inflammation or changes in body composition.

**Table 3:** Effects of human fecal microbiota transplant (FMT) of trained and non-trained bariatric women on body weight and biochemical and inflammatory parameters in SPF female mice with diet-induced obesity.

Variables	rRYGB + ET (*n* = 11)	rRYGB (*n* = 12)	rLEAN (*n* = 12)	*P*-value Group	*P*-value Group x Time
Body weight (g)					0.18
PRE_FMT_^a^	25.39 ± 2.85	25.19 ± 4.44	24.73 ± 3.99	–	
POST_FMT_^b^	33.06 ± 5.59	34.29 ± 7.99	30.98 ± 4.90	–	
Fasting glucose (mg/dL)					0.71
PRE_FMT_^a^	169.50 ± 17.70	161.50 ± 14.34	175.16 ± 19.30	–	
POSTFMT^b^	196.54 ± 21.79	186.00 ± 19.86	192.83 ± 28.28	–	
AUC_glucose_ (mg/dL x min)					0.88
PRE_FMT_	468.72 ± 101.45	425.31 ± 71.64	427.32 ± 42.07	–	
POST_FMT_	470.11 ± 66.69	448.81 ± 44.40	441.52 ± 45.47	–	
**POST**_**FMT**_					
Fasting insulin (μU/mL)	1.06 (0.50)^#^	2.29 (1.45)*	1.36 ± 0.89	0.02	–
HOMA-IR	0.50 (0.21)^#^	1.04 (0.63)*	0.64 ± 0.45	0.03	–
Adiposity (g fat/g bw)	0.92 (0.24)	1.14 (0.59)	0.87 ± 0.24	0.22	–
TNF (mg/dL)	1.33 ± 0.80	1.74 ± 2.21	1.75 ± 1.58	0.81	–
IL-6 (mg/dL)	18.48 ± 19.13	14.60 ± 14.68	11.15 ± 9.04	0.52	–
IL-10 (mg/dL)	20.48 ± 25.53	13.41 ± 12.03	19.62 ± 35.86	0.79	–
IL-1ß (mg/dL)	7.61 ± 10.64	4.48 ± 8.71	3.49 ± 7.49	0.58	–

Data as mean ± standard deviation, level of significance (*p*-value; group effect), and level of significance (interaction effect) calculated using a mixed model for repeated measures. Different superscript letters denote significantly different values for main effect of time (*p* ≤ 0.05).

*Means *p* ≤ 0.05 for between-group comparisons (*vs*. LEAN); #means *p* ≤ 0.05 for between-group comparisons (*vs*. RYGB).

**Fig. 4:**
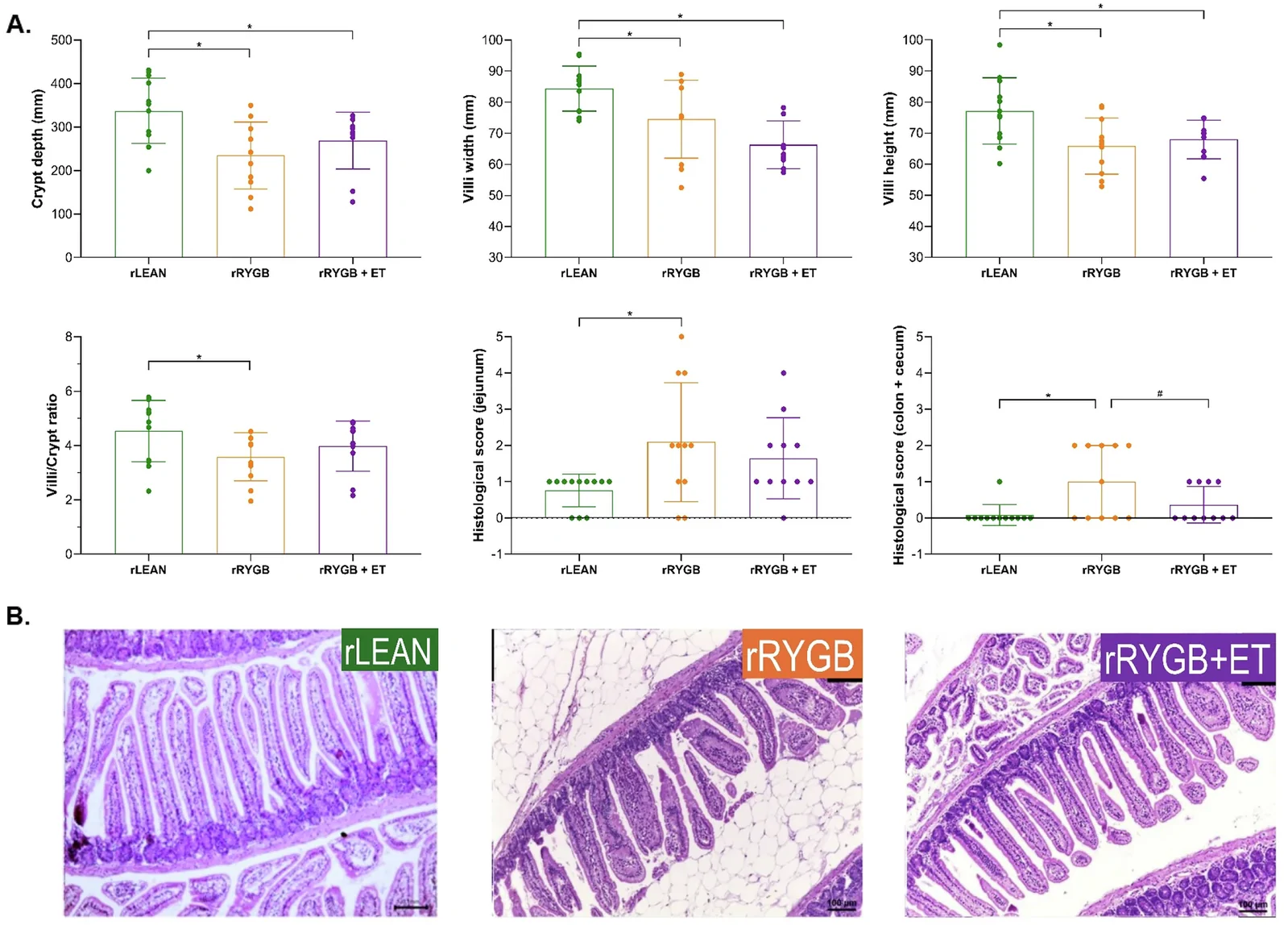
Intestinal morphology following FMT from trained vs. non-trained donors. **A** Morphological parameters; **B** Representative images. Data are presented as mean ± standard deviation. * indicates significant between-group differences.

Histological analysis showed that rRYGB displayed impaired intestinal morphology scores compared with rLEAN, whereas rRYGB+ET mice showed partial preservation of villus-to-crypt ratio and inflammatory histological scores compared with mice receiving RYGB microbiota (Fig. 4). Together, these findings suggest that microbiota from exercise-trained patients may contribute to protection against diet-induced insulin resistance and intestinal atrophy, without detectable changes in adiposity or inflammatory profile.

### Exercise-conditioned microbiota enriched butyrate- and bile acid–modulating genera in mice with obesity

After FMT, rRYGB showed an increase in α-diversity (Shannon index) and a reduction in relative dominance, whereas no between-group differences or significant changes occurred in rRYGB+ET or rLEAN (Supplementary Fig. 6). β-diversity analyses indicated similar overall shifts in microbial community composition over time among groups. However, increases in community dispersion suggested that POST_FMT_ microbial profiles were not only altered relative to baseline but also became more heterogeneous across individual recipients (Supplementary Fig. 7).

Differential abundance analysis revealed no between-group differences across time. Within-group changes included increases in *Butyricimonas* across all groups. In rRYGB, *Akkermansia*, *Butyricicoccus*, and *Olsenella* decreased, while *Christensenella*, *Anaerofilum*, *Enterocloster*, and *Desulfovibrio* increased. In contrast, rRYGB+ET exhibited decreases in *Aminipila*, *Paludihabitans*, and *Ruthenibacterium*, alongside increases in *Roseburia*, *Muriventricola*, and *Odoribacter*. *Massiliimalia* increased in both rRYGB+ET and rLEAN, while *Anaerotruncus* decreased and *Holdemania* and *Collinsella* increased in rLEAN (Supplementary Fig. 8). These compositional patterns are notable given that *Roseburia*, *Muriventricola*, *Odoribacter* are associated with carbohydrate fermentation, SCFA synthesis, and bile acid metabolism [35, 36]. Conversely, the loss of *Akkermansia*, *Butyricicoccus*, and *Olsenella* in rRYGB is consistent with a less favorable metabolic and inflammatory profile [35, 37]. Notably, fecal SCFA concentrations did not differ between groups (Supplementary Table 4). Thus, the metabolic differences observed in rRYGB+ET were not accompanied by detectable differences in bulk fecal SCFA levels, suggesting that other microbial or host-related mechanisms may be involved.

## Discussion

We investigated whether exercise-related microbial changes are associated with metabolic adaptations in women undergoing Roux-en-Y gastric bypass (RYGB) by combining a randomized controlled exercise intervention trial in women post-surgery with a human-to-mouse fecal microbiota transplantation (FMT) experiment in high-fat-fed mice. Six months of structured exercise training did not enhance changes in body composition but was associated with improvements in insulin sensitivity and lipid profile. These benefits were accompanied by increased microbial α-diversity and distinct compositional shifts, including preservation of taxa previously associated with short-chain fatty acid (SCFA) metabolism and lower relative abundance of genera considered potential pathobionts. Importantly, transplantation of microbiota from exercising RYGB patients into recipient mice with obesity was associated with lower fasting insulin and improved HOMA-IR compared with mice receiving microbiota from non-exercising RYGB donors, independent of body composition. Together, these findings suggest that exercise may promote specific gut microbial configurations that contribute to metabolic improvements beyond those conferred by surgery alone.

Consistent with previous studies [11–14], RYGB was associated with enrichment of genera involved in the fermentation of simple carbohydrates, lactate, and amino acids, including *Veillonella*, *Granulicatella*, *Streptococcus*, *Gemella*, *Fusobacterium*, and facultative anaerobes such as *Escherichia/Shigella*. Their expansion may reflect 1) microbial adaptation to the altered post-surgical gut environment, characterized by modified nutrient flow, bile acids, and pH [38–40]; and 2) the reduction in dietary intake, including decreases in total energy and fiber consumption, which favors a shift toward microbial networks relying on lactate and other rapidly fermentable substrates [41]. In parallel, several genera known for fermenting complex carbohydrates and fiber and commonly associated with SCFA metabolism [39] were reduced post-RYGB, including *Anaerostipes, Flavonifractor* and *Turicibacter*. The reduced availability of fermentable substrates such as carbohydrates and fibers may help to explain their decline despite the overall metabolic improvements observed after surgery.

Exercise training increased gut microbiota α-diversity, consistent with previous findings [23], and attenuated the reduction taxa associated with carbohydrate fermentation and SCFA production, such as *Anaerostipes, Flavonifractor* and *Turicibacter*. These shifts may indicate a microbial community with greater potential for carbohydrate fermentation and butyrate production, metabolites that have been linked to epithelial integrity, gut-derived gluconeogenesis, and hepatic insulin sensitivity [42]. However, fecal butyrate concentrations did not differ between groups in the present study. It is important to note that fecal SCFAs represent only a small fraction (5%) of total SCFA production because most are rapidly absorbed in the colon [43, 44]. Thus, fecal concentrations may not fully reflect intestinal production or host exposure. Additional studies incorporating isotope tracing or direct measurements of circulating metabolites would be required to clarify the contribution of SCFAs to the metabolic effects observed after surgery combined with exercise.

Exercise training also attenuated the post-surgical increase in *Escherichia/Shigella*. Members of this genus have been linked to increased endotoxin exposure, a canonical driver of hepatic insulin resistance [45]. Thus, their reduction may therefore reflect a microbial configuration associated with improved metabolic homeostasis, without altering systemic inflammatory markers. Concurrently, exercise was associated with increased abundance of beneficial genera such as *Rothia, Jutongia*, and *Granulicatella*, which have been linked to nitrate-reducing pathways and metabolic health in prior studies [33]. Together, these microbial shifts may explain the greater improvements in fasting insulin, C-peptide, HOMA-IR, HDL, and triglycerides in the RYGB + ET group. Although these associations are consistent with the greater improvements in fasting insulin, C-peptide, HOMA-IR, HDL, and triglycerides observed in the RYGB + ET group [46–49], the functional implications of these microbial shifts should be interpreted cautiously because microbial metabolites and endotoxin-related markers were not directly measured.

The FMT experiment provides complementary evidence supporting a potential contribution of the microbiota to metabolic regulation. Mice colonized with microbiota from RYGB + ET donors exhibited lower fasting insulin and improved hepatic insulin sensitivity compared with those receiving RYGB microbiota, despite similar body weight and inflammatory profiles. In addition, partial preservation of intestinal architecture in rRYGB+ET mice may indicate greater epithelial resilience, which could influence nutrient absorption and metabolic regulation [50, 51]. Previous studies have shown that exercise can modulate intestinal morphology and metabolic outcomes in experimental models [52]. Nevertheless, because gut barrier integrity and endotoxin exposure were not directly assessed in the present study, these mechanisms remain to be determined.

Microbial changes in recipient mice were consistent with this interpretation. rRYGB mice displayed reductions in *Akkermansia*, a genus frequently associated with improved glucose homeostasis, gut barrier function, and reduced inflammation [37], as well as decreases in *Butyricicoccus* and *Olsenella*, both linked to fiber fermentation and SCFA metabolism [35]. Conversely, rRYGB+ET showed increased *Roseburia* and *Muriventricola*, taxa associated with carbohydrate fermentation and butyrate production [53], and *Odoribacter*, which has been linked to bile acid metabolism and SCFA production [54]. Although these patterns align with the metabolic differences observed between groups, functional conclusions remain inferential and should be interpreted within the context of taxonomic associations.

Unlike the clinical trial, α-diversity increased in rRYGB but not in rRYGB+ET or rLEAN. Although somewhat unexpected, several studies have reported inconsistent relationships between bacterial diversity and metabolic outcomes in bariatric patients [55, 56]. Similarly, Anhê et al. [17] observed improved glucose control without changes in α-diversity in mice receiving FMT from bariatric patients. These findings suggest that microbial composition and function may be more relevant to metabolic regulation than diversity per se, although further mechanistic studies are needed to clarify this relationship.

RYGB and RYGB + ET showed comparable weight loss, fat mass reduction, food intake changes and systemic inflammation, suggesting that the superior metabolic outcomes observed in the exercise group were unlikely to be explained by differences in food intake, anthropometric or inflammatory parameters. Likewise, in the murine FMT model, rRYGB+ET recipients showed no differences in body mass, adipose tissue inflammation, or systemic inflammatory markers compared with rRYGB. These cross-species findings are consistent with a potential contribution of microbiota-related mechanisms to the metabolic improvements observed with exercise, although interactions between microbial, host, and environmental factors cannot be fully disentangled.

This study provides a translational framework linking exercise, microbiota, and metabolic recovery after bariatric surgery. However, several limitations should be considered. These include the modest sample size and the inclusion of women only, which may limit generalizability. Some findings were interpreted as trends (0.05 < *p* ≤ 0.10) and should therefore be considered exploratory. In the murine FMT model, colonization efficiency reached approximately 58%. Although this level of engraftment supports the interpretation that donor microbial communities contributed to the observed metabolic differences, the use of antibiotic-treated SPF mice may still allow residual host microbiota to influence colonization dynamics. Future studies using germ-free models could provide an even more controlled framework to investigate microbiota-mediated mechanisms and maximize donor microbial engraftment. The use of pooled donor material for FMT may also be considered a limitation. While pooling allowed the generation of a representative microbial inoculum for each clinical group, it precluded the evaluation of donor-specific variability and prevented the identification of individual microbial features that might be associated with the observed metabolic effects in recipient mice. Additional limitations include the absence of bile acid profiling and other microbial metabolites (e.g., trimethylamine, TMA) [56], as well as the lack of direct measurements of gut barrier integrity or endotoxin burden. Consequently, the functional pathways linking microbial changes to host metabolism remain to be clarified in future studies using integrated metagenomic, metabolomic, and physiological approaches. Finally, as expected after bariatric surgery, several medications decreased over time in both groups, including metformin, which is known to influence gut microbiota composition [57]. However, because medication reductions occurred similarly in RYGB and RYGB + ET participants and no antibiotics were used during the study period, medication changes are unlikely to account for the exercise-specific microbial alterations observed.

In summary, structured exercise following bariatric surgery was associated with improved metabolic outcomes and distinct gut microbiota configurations. The accompanying FMT experiments suggest that microbiota derived from exercising hosts may contribute to aspects of metabolic regulation following surgery. Although the specific microbial metabolites and host pathways responsible remain to be determined, these findings support a potential role for the gut microbiota in mediating some of the metabolic benefits of exercise in the post-bariatric setting.

## Supplementary information

Supplementary Table 1

Supplementary Table 2

Supplementary Table 3

Supplementary Table 4

Supplementary information

Supplementary Figure 1

Supplementary Figure 2

Supplementary Figure 3

Supplementary Figure 4

Supplementary Figure 5

Supplementary Figure 6

Supplementary Figure 7

Supplementary Figure 8

## Data Availability

All sequencing data have been deposited in the National Center for Biotechnology Information (NCBI) at the Sequence Read Archive (SRA) under the BioProject accession number SRA: PRJNA1364303. The anonymized data generated and analyzed during the current study are available from the Chief Investigator (corresponding author) upon reasonable request. Pending approval by all authors, data will be shared via a secure online platform.
