# Supplementary material for: Exercise reprograms the gut microbiota to enhance metabolic outcomes after bariatric surgery: a translational, cross-species study
Source: Int J Obes (Lond). 2026 Jun 4;50(10):2113–25. doi: 10.1038/s41366-026-02121-3 (PMC13722583; doi:10.1038/s41366-026-02121-3)
Supplement: Supplementary file 1 [file 41366_2026_2121_MOESM10_ESM.docx]

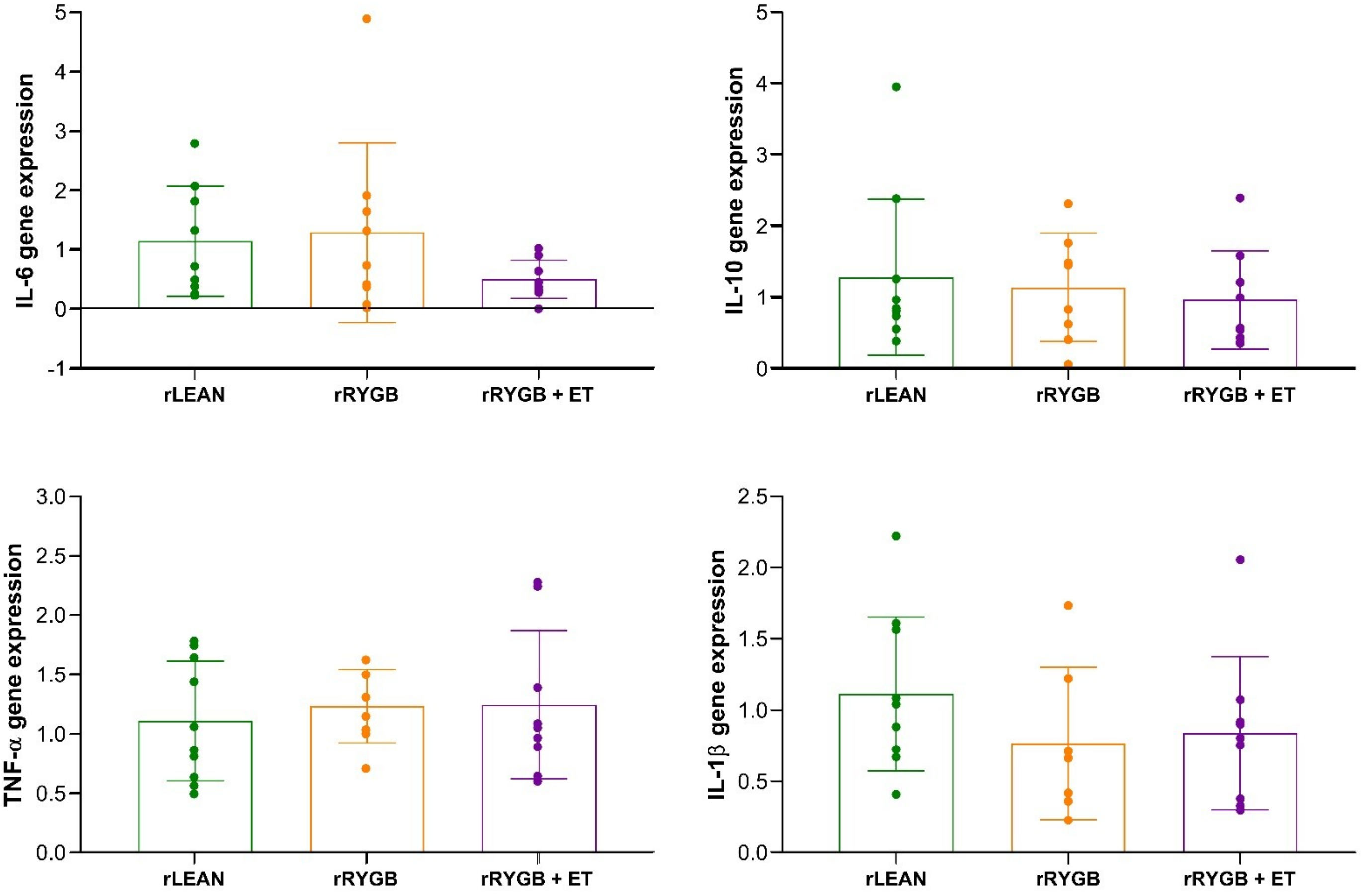

IL-6 gene expression
IL-10 gene expression
TNF-α gene expression
IL-1β gene expression
rLEAN
rRYGB
rRYGB + ET
