# Supplementary material for: Exercise reprograms the gut microbiota to enhance metabolic outcomes after bariatric surgery: a translational, cross-species study
Source: Int J Obes (Lond). 2026 Jun 4;50(10):2113–25. doi: 10.1038/s41366-026-02121-3 (PMC13722583; doi:10.1038/s41366-026-02121-3)
Supplement: Supplementary file 2 [file 41366_2026_2121_MOESM11_ESM.docx]

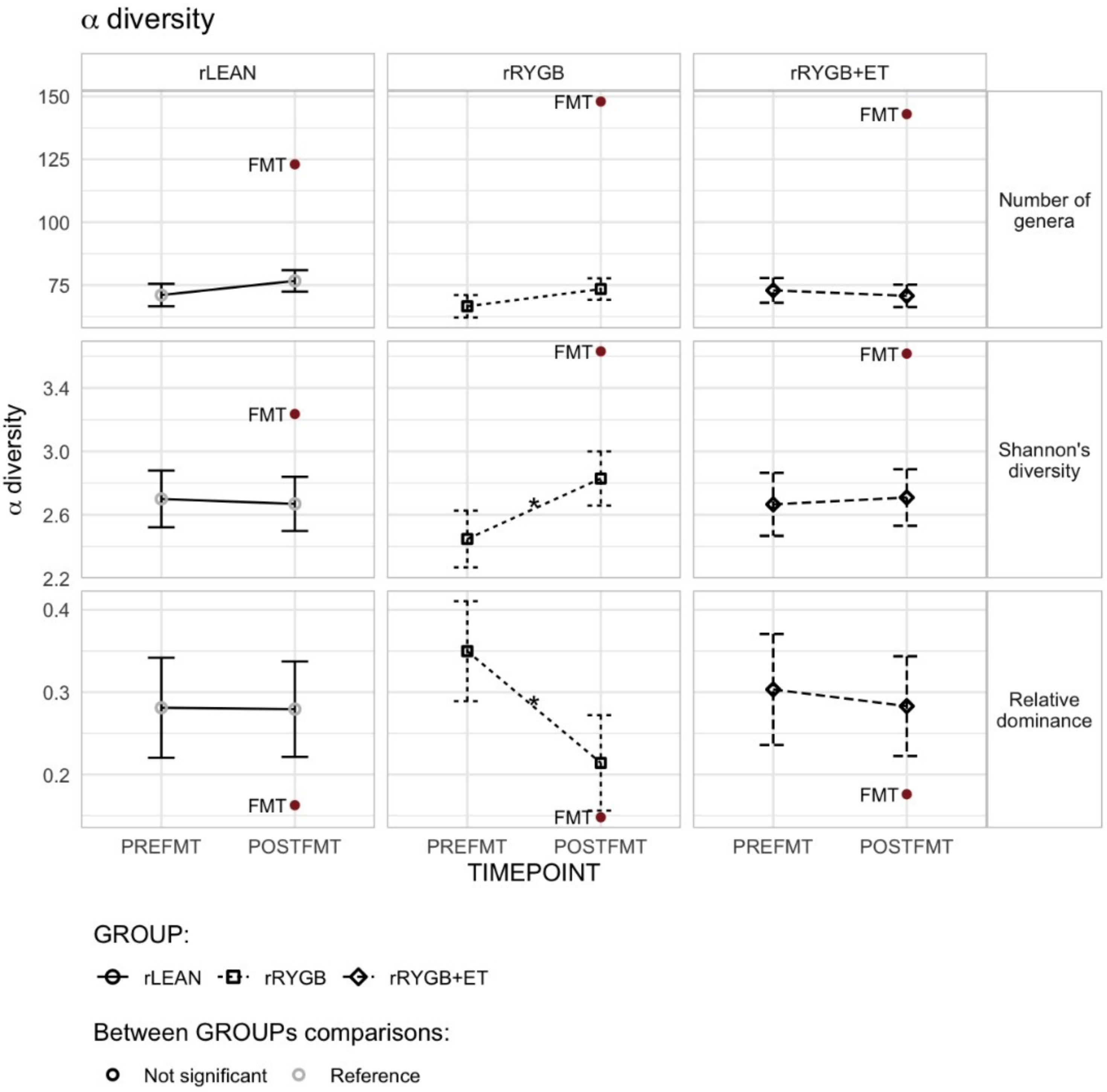

α diversity
rLEAN
rRYGB
rRYGB+ET
Number of genera
Shannon's diversity
Relative dominance
FMT
*
α diversity
150
125
100
75
3.4
3.0
2.6
2.2
0.4
0.3
0.2
PREFMT
POSTFMT
TIMEPOINT
GROUP:
rLEAN
rRYGB
rRYGB+ET
Between GROUPs comparisons:
Not significant
Reference
