# Supplementary material for: Exercise reprograms the gut microbiota to enhance metabolic outcomes after bariatric surgery: a translational, cross-species study
Source: Int J Obes (Lond). 2026 Jun 4;50(10):2113–25. doi: 10.1038/s41366-026-02121-3 (PMC13722583; doi:10.1038/s41366-026-02121-3)
Supplement: Supplementary file 3 [file 41366_2026_2121_MOESM12_ESM.docx]

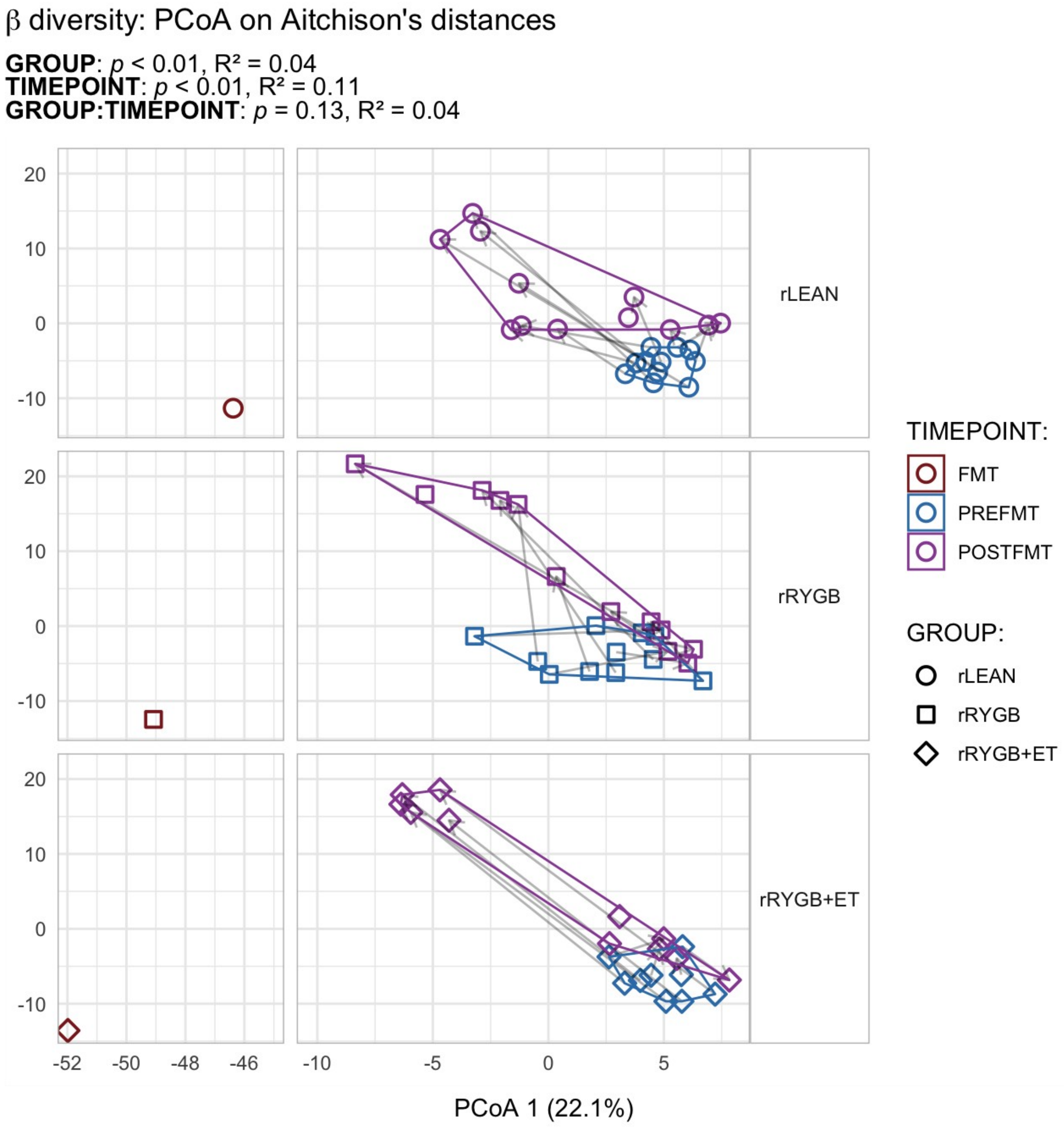

β diversity: PCoA on Aitchison's distances
GROUP: p < 0.01, R² = 0.04
TIMEPOINT: p < 0.01, R² = 0.11
GROUP:TIMEPOINT: p = 0.13, R² = 0.04
rLEAN
rRYGB
rRYGB+ET
PCoA 2 (13.4%)
PCoA 1 (22.1%)
TIMEPOINT:
FMT
PREFMT
POSTFMT
GROUP:
rLEAN
rRYGB
rRYGB+ET
