# Supplementary material for: Exercise reprograms the gut microbiota to enhance metabolic outcomes after bariatric surgery: a translational, cross-species study
Source: Int J Obes (Lond). 2026 Jun 4;50(10):2113–25. doi: 10.1038/s41366-026-02121-3 (PMC13722583; doi:10.1038/s41366-026-02121-3)
Supplement: Supplementary file 4 [file 41366_2026_2121_MOESM13_ESM.docx]

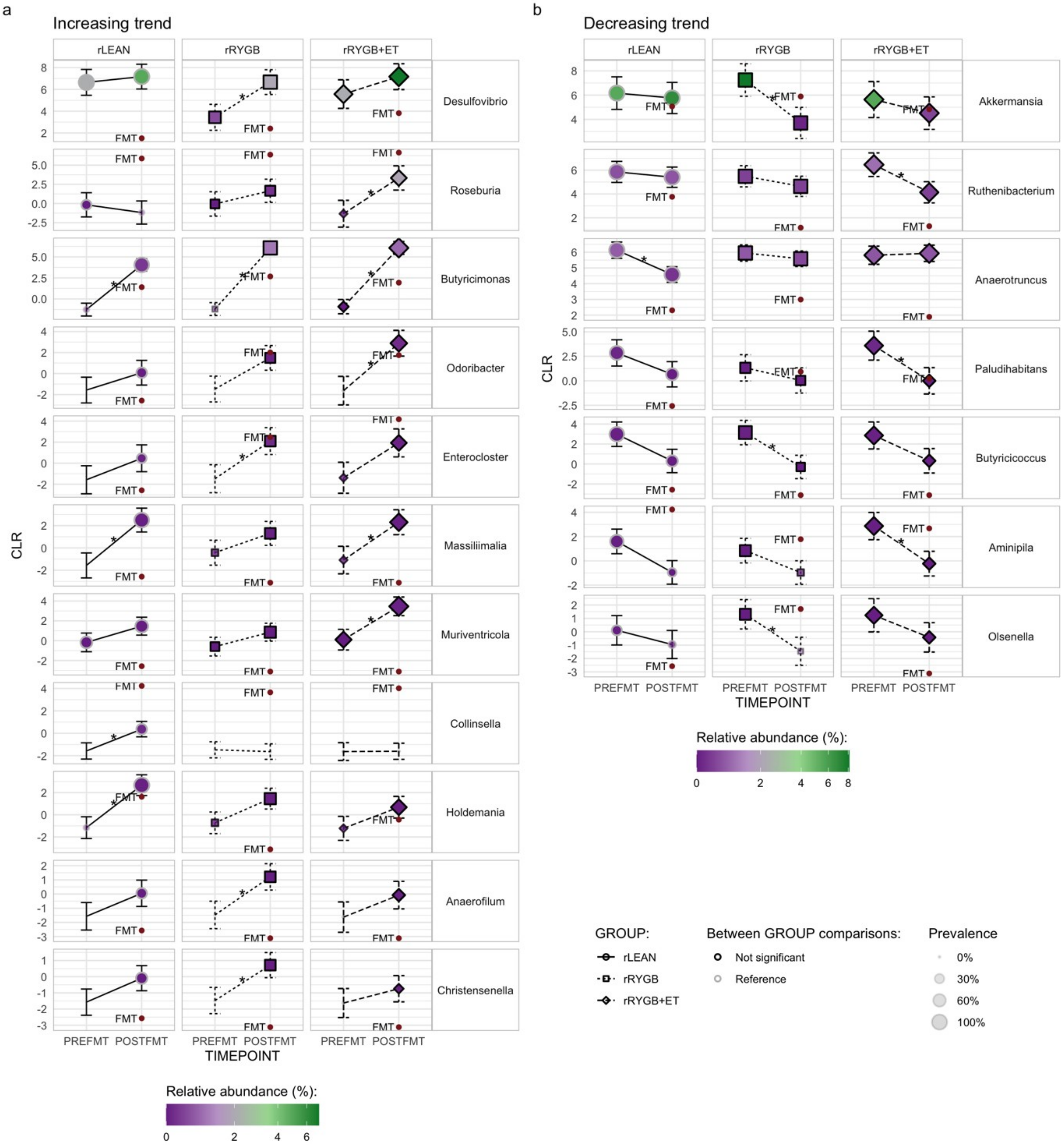

a
Increasing trend
rLEAN
rRYGB
rRYGB+ET
Desulfovibrio
Roseburia
Butyricimonas
Odoribacter
Enterocloster
Massiliimalia
Muriventricola
Collinsella
Holdemania
Anaerofilum
Christensenella
FMT
CLR
PREFMT
POSTFMT
TIMEPOINT
Relative abundance (%):
b
Decreasing trend
Akkermansia
Ruthenibacterium
Anaerotruncus
Paludihabitans
Butyricicoccus
Aminipila
Olsenella
GROUP:
rLEAN
rRYGB
rRYGB+ET
Between GROUP comparisons:
Not significant
Reference
Prevalence
0%
30%
60%
100%
