# Supplementary material for: Exercise reprograms the gut microbiota to enhance metabolic outcomes after bariatric surgery: a translational, cross-species study
Source: Int J Obes (Lond). 2026 Jun 4;50(10):2113–25. doi: 10.1038/s41366-026-02121-3 (PMC13722583; doi:10.1038/s41366-026-02121-3)
Supplement: Supplementary file 5 [file 41366_2026_2121_MOESM6_ESM.docx]

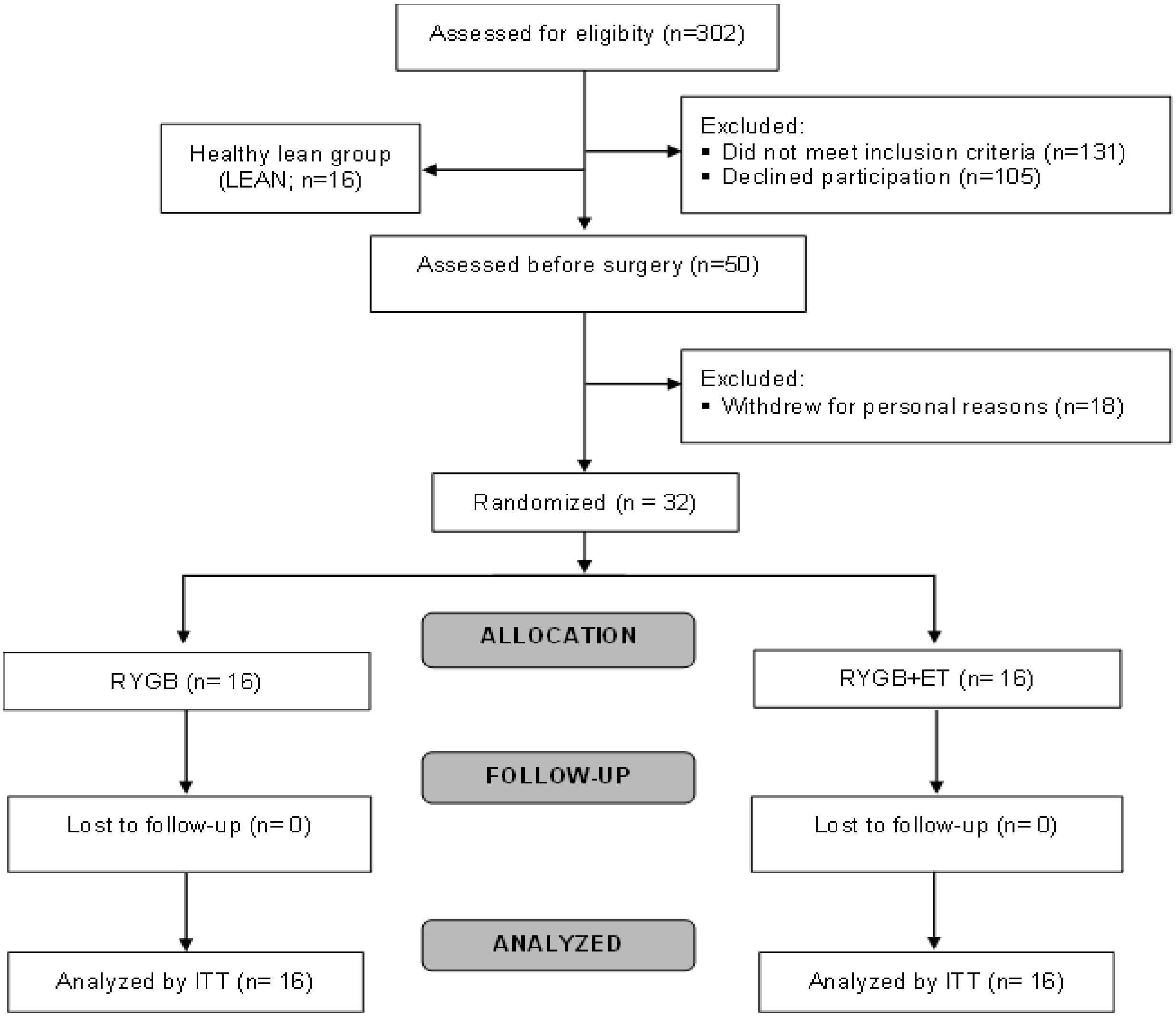
Assessed for eligibity (n=302)
Healthy lean group (LEAN; n=16)
Excluded:
Did not meet inclusion criteria (n=131)
Declined participation (n=105)
Assessed before surgery (n=50)
Excluded:
Withdrew for personal reasons (n=18)
Randomized (n = 32)
ALLOCATION
RYGB (n= 16)
RYGB+ET (n= 16)
FOLLOW-UP
Lost to follow-up (n= 0)
Lost to follow-up (n= 0)
ANALYZED
Analyzed by ITT (n= 16)
Analyzed by ITT (n= 16)
