# Supplementary material for: Exercise reprograms the gut microbiota to enhance metabolic outcomes after bariatric surgery: a translational, cross-species study
Source: Int J Obes (Lond). 2026 Jun 4;50(10):2113–25. doi: 10.1038/s41366-026-02121-3 (PMC13722583; doi:10.1038/s41366-026-02121-3)
Supplement: Supplementary file 6 [file 41366_2026_2121_MOESM7_ESM.docx]

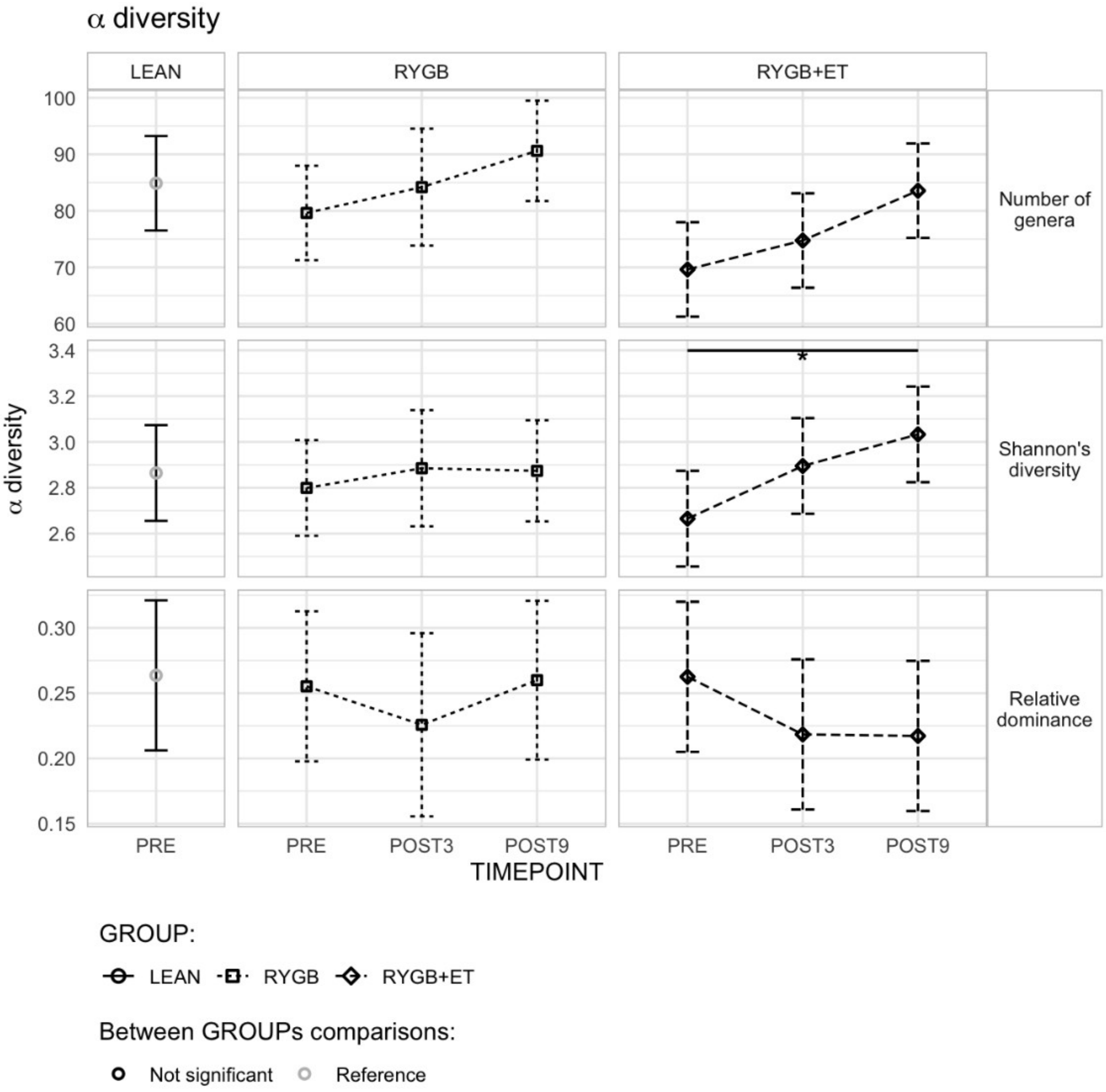

α diversity
LEAN
RYGB
RYGB+ET
100
90
80
70
60
3.4
3.2
3.0
2.8
2.6
0.30
0.25
0.20
0.15
Number of genera
Shannon's diversity
Relative dominance
α diversity
*
PRE
POST3
POST9
TIMEPOINT
GROUP:
LEAN
RYGB
RYGB+ET
Between GROUPs comparisons:
Not significant
Reference
