# Supplementary material for: Exercise reprograms the gut microbiota to enhance metabolic outcomes after bariatric surgery: a translational, cross-species study
Source: Int J Obes (Lond). 2026 Jun 4;50(10):2113–25. doi: 10.1038/s41366-026-02121-3 (PMC13722583; doi:10.1038/s41366-026-02121-3)
Supplement: Supplementary file 7 [file 41366_2026_2121_MOESM8_ESM.docx]

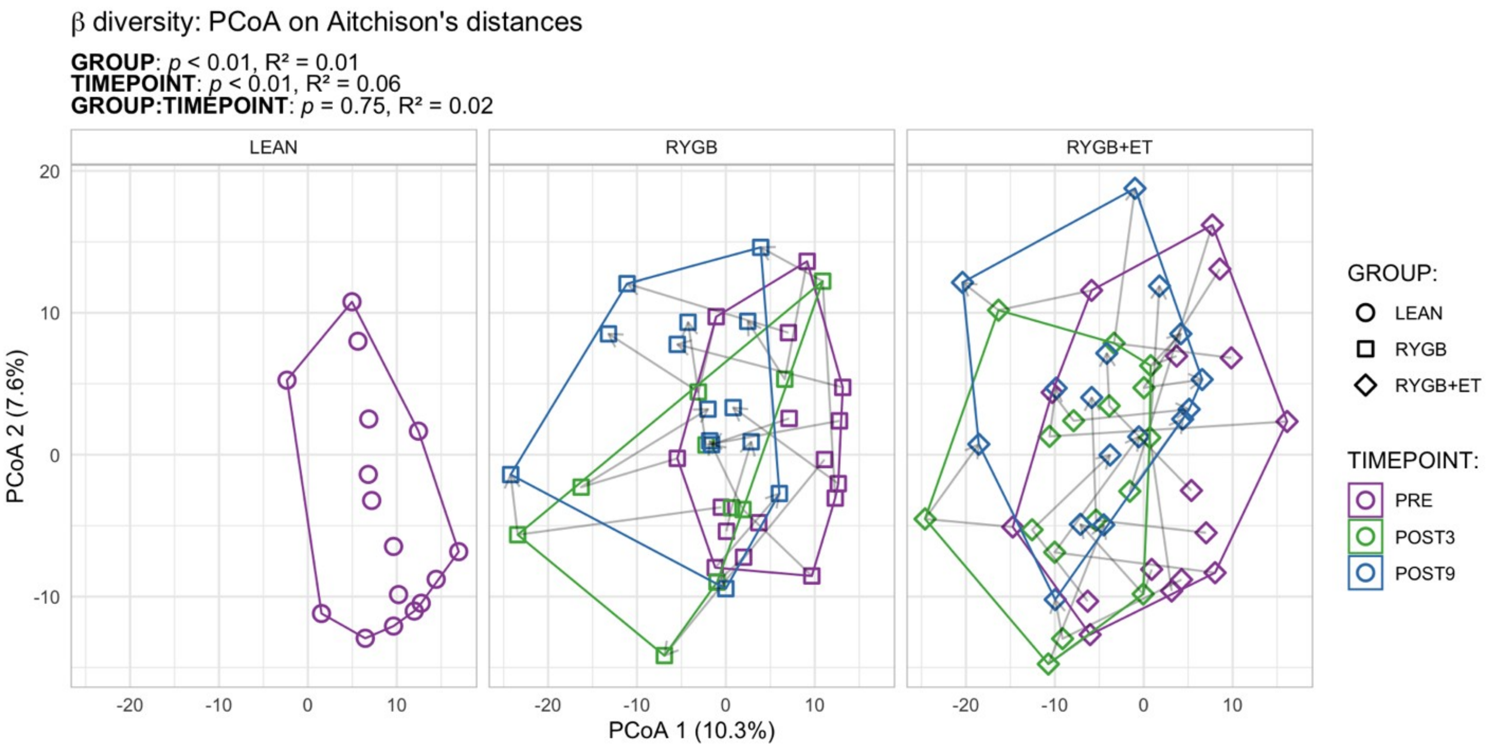
β diversity: PCoA on Aitchison's distances
GROUP: p < 0.01, R² = 0.01
TIMEPOINT: p < 0.01, R² = 0.06
GROUP:TIMEPOINT: p = 0.75, R² = 0.02
LEAN
RYGB
RYGB+ET
PCoA 2 (7.6%)
PCoA 1 (10.3%)
GROUP:
LEAN
RYGB
RYGB+ET
TIMEPOINT:
PRE
POST3
POST9
