# Supplementary material for: Exercise reprograms the gut microbiota to enhance metabolic outcomes after bariatric surgery: a translational, cross-species study
Source: Int J Obes (Lond). 2026 Jun 4;50(10):2113–25. doi: 10.1038/s41366-026-02121-3 (PMC13722583; doi:10.1038/s41366-026-02121-3)
Supplement: Supplementary file 8 [file 41366_2026_2121_MOESM9_ESM.docx]

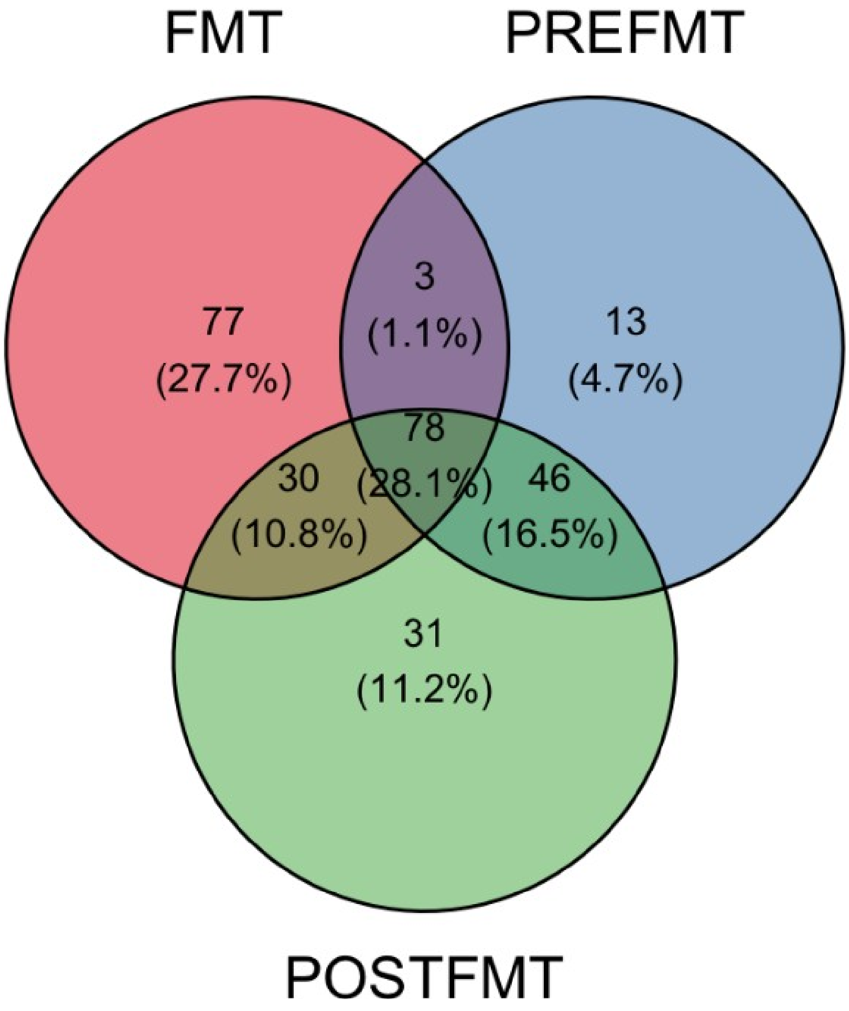
FMT
PREFMT
77
(27.7%)
3
(1.1%)
13
(4.7%)
78
(28.1%)
30
(10.8%)
46
(16.5%)
31
(11.2%)
POSTFMT
