# Supplementary material for: Exercise reprograms the gut microbiota to enhance metabolic outcomes after bariatric surgery: a translational, cross-species study
Source: Int J Obes (Lond). 2026 Jun 4;50(10):2113–25. doi: 10.1038/s41366-026-02121-3 (PMC13722583; doi:10.1038/s41366-026-02121-3)
Supplement: Supplementary file 5 [file 41366_2026_2121_MOESM1_ESM.docx]

**Supplementary Table 1.** Characteristics of medication among bariatric patients.

| **MEDICATIONS** | **RYGB (n= 16)** | | | **RYGB + ET (n= 16)** | | |
| --- | --- | --- | --- | --- | --- | --- |
|  | **PRE** | **POST3** | **POST9** | **PRE** | **POST3** | **POST9** |
| Antibiotics | 0 (0.0) | 0 (0.0) | 0 (0.0) | 0 (0.0) | 0 (0.0) | 0 (0.0) |
| Anxiolytics | 0 (0.0) | 0 (0.0) | 0 (0.0) | 4 (25.0) | 2 (12.5) | 0 (0.0) |
| Antiepileptic | 2 (12.5) | 2 (12.5) | 2 (12.5) | 0 (0.0) | 0 (0.0) | 0 (0.0) |
| Alpha-2 Adrenergic Agonist | 2 (12.5) | 2 (12.5) | 0 (0.0) | 0 (0.0) | 0 (0.0) | 0 (0.0) |
| A-II Receptor Antagonist | 1 (6.3) | 1 (6.3) | 0 (0.0) | 2 (12.5) | 2 (12.5) | 0 (0.0) |
| Calcium Blocker | 0 (0.0) | 0 (0.0) | 0 (0.0) | 2 (12.5) | 2 (12.5) | 0 (0.0) |
| Diuretic | 4 (25.0) | 4 (25.0) | 2 (12.5) | 7 (43.8) | 4 (25.0) | 0 (0.0) |
| ACE Inhibitors | 0 (0.0) | 0 (0.0) | 0 (0.0) | 1 (6.3) | 1 (6.3) | 0 (0.0) |
| Insulin | 0 (0.0) | 0 (0.0) | 0 (0.0) | 0 (0.0) | 0 (0.0) | 0 (0.0) |
| Metformin | 7 (43.8) | 7 (43.8) | 2 (12.5) | 11 (68.8) | 7 (43.8) | 0 (0.0) |

Data expressed as absolute and relative frequency n (%). ACE: Angiotensin Converting Enzyme.
