# Supplementary material for: Exercise reprograms the gut microbiota to enhance metabolic outcomes after bariatric surgery: a translational, cross-species study
Source: Int J Obes (Lond). 2026 Jun 4;50(10):2113–25. doi: 10.1038/s41366-026-02121-3 (PMC13722583; doi:10.1038/s41366-026-02121-3)
Supplement: Supplementary file 6 [file 41366_2026_2121_MOESM2_ESM.docx]

**Supplementary Table 2.** Effects of exercise training on anthropometric measurements, body composition, physical capacity and levels, and food intake in women undergoing bariatric surgery

|  | **RYGB**  **(n= 16)** | **RYGB+ET**  **(n= 16)** | **Difference**  **(IC - 95%)** | **P-value** | **P-value**  **Group x Time** |
| --- | --- | --- | --- | --- | --- |
| Body weight (kg) |  |  |  |  | 0.371 |
| PRE^a^ | 123.18±20.76 | 117.48±19.53 | 1.17 (-4.77 to 7.12) | 0.69 |  |
| POST3^b^ | 94.93±18.72 | 93.29±16.33 | -3.97 (-10.39 to 2.43) | 0.22 |  |
| POST9^c^ | 81.88±15.88 | 79.88±16.56 | -3.03 (-9.18 to 3.12) | 0.32 |  |
| ∆ change | -41.58±12.07 | -37.59±12.76 | -1.89 (-9.72 to 5.93) | 0.62 |  |
| BMI (kg/m^2^) |  |  |  |  | 0.566 |
| PRE^a^ | 38.61±6.45 | 37.43±6.46 | 0.43 (-2.08 to 2.94) | 0.73 |  |
| POST3^b^ | 28.83±5.12 | 29.02±4.45 | -1.29 (-4.01 to 1.41) | 0.34 |  |
| POST9^c^ | 24.94±4.37 | 24.86±4.61 | -0.83 (-3.43 to 1.76) | 0.56 |  |
| ∆ change | -13.83±5.44 | -12.56±5.62 | -0.45 (-3.42 to 2.50) | 0.75 |  |
| Waist circumference (cm) |  |  |  |  | 0.795 |
| PRE^a^ | 119.23±11.45 | 122.83±11.96 | -0.64 (-5.96 to 4.67) | 0.80 |  |
| POST3^b^ | 99.32±9.61 | 106.97±11.72 | -2.43 (-8.22 to 3.35) | 0.40 |  |
| POST9^c^ | 89.96±8.92 | 94.93±13.36 | -0.22 (-5.67 to 5.21) | 0.93 |  |
| ∆ change | -27.21±8.41 | -27.90±12.75 | -1.61 (-9.60 to 6.38) | 0.68 |  |
| Fat Mass (g) |  |  |  |  | 0.845 |
| PRE^a^ | 62.47±14.35 | 61.24±11.82 | 0.20 (-3.97 to 4.37) | 0.92 |  |
| POST3^b^ | 45.67±12.57 | 43.65±9.56 | -1.55 (-6.41 to 3.29) | 0.52 |  |
| POST9^c^ | 33.78±10.27 | 30.31±13.18 | -0.80 (-5.31 to 3.70) | 0.72 |  |
| ∆ change | -31.62±9.27 | -30.09±10.62 | -1.52 (-9.33 to 6.27) | 0.94 |  |
| Lean Mass (g) |  |  |  |  | **0.011** |
| PRE | 55.11±6.65 | 52.18±7.04 | 0.23 (-1.50 to 1.97) | 0.78 |  |
| POST3 | 48.45±7.46* | 45.36±5.32* | -1.38 (-3.40 to 0.61) | 0.17 |  |
| POST9 | 47.76±5.15* | 47.17±7.87* | -3.27 (-5.15 to -1.40) | **0.001** |  |
| ∆ change | -8.49±4.14 | -4.85±2.60 | -3.00 (-5.69 to -0.32) | **0.02** |  |
| Visceral Adipose Tissue (cm^3^) | |  |  |  | 0.279 |
| PRE^a^ | 2.05±0.65 | 2.42±0.82 | -0.07 (-0.27 to 0.12) | 0.44 |  |
| POST3^b^ | 1.52±0.57 | 1.48±0.54 | 0.10 (-0.12 to 0.33) | 0.36 |  |
| POST9^c^ | 0.94±0.29 | 0.96±0.73 | 0.15 (-0.06 to 0.36) | 0.16 |  |
| ∆ change | -1.22±0.54 | -1.42±0.37 | 0.10 (-0.16 to 0.38) | 0.42 |  |
| VO_2peak_ (ml/Kg/min) |  |  |  |  | **0.009** |
| PRE | 15.52±3.38 | 14.04±2.85 | 0.33 (-3.04 to 3.71) | 0.99 |  |
| POST3 | 20.20±4.90* | 17.50±3.56* | 1.12 (-3.02 to 5.26) | 0.96 |  |
| POST9 | 20.39±2.66* | 22.13±4.89*^$^ | -3.86 (-7.82 to 0.10) | **0.06** |  |
| ∆ change | 3.45±3.98 | 8.12±3.70 | -3.96 (-7.69 to -0.22) | **0.03** |  |
| 1-RM leg-press (Kg) |  |  |  |  | **<0.0001** |
| PRE | 210.00±56.24 | 191.25±53.81 | 3.53 (-25.37 to 32.44) | 0.99 |  |
| POST3 | 136.36±54.98* | 141.56±42.19* | -22.59 (-54.69 to 9.51) | 0.31 |  |
| POST9 | 125.71±48.80* | 176.25±58.62 | -70.55 (-100.60 to -40.52) | **<0.0001** |  |
| ∆ change | -90.00±51.14 | -15.00±31.35 | -67.96 (-97.94 to -37.99) | **<0.0001** |  |
| 1-RM bench-press (Kg) |  |  |  |  | **0.006** |
| PRE | 38.56±8.60 | 37.60±9.88 | 0.27 (-4.86 to 5.44) | 0.99 |  |
| POST3 | 26.91±7.35* | 26.75±6.48* | -0.67 (-6.39 to 5.04) | 0.99 |  |
| POST9 | 25.14±7.47* | 30.75±7.24* | -7.21 (-12.57 to -1.86) | **0.003** |  |
| ∆ change | -14.78±8.43 | -6.93±7.70 | -7.85 (-14.00 to -1.70) | **0.01** |  |
| Sedentary time (min/day) |  |  |  |  | 0.10 |
| PRE | 558.78±210.51 | 593.61±85.34 | -26.83 (-183.5 to 129.8) | 0.99 |  |
| POST3 | 548.59±188.86 | 541.38±103.15 | 42.13 (-131.2 to 215.5) | 0.97 |  |
| POST9 | 571.85±119.73 | 433.68±109.27* | 150.30 (-9.88 to 310.5) | **0.08** |  |
| ∆ change | 43.07±270.34 | -149.53±148.49 | 192.60 (14.10 to 371.1) | **0.03** |  |
| LPA (min/day) |  |  |  |  | **0.008** |
| PRE | 79.71±34.45 | 78.09±24.27 | 0.67 (-43.02 to 44.36) | 0.99 |  |
| POST3 | 77.00±40.78 | 89.60±32.30 | -16.81 (-65.34 to 31.72) | 0.90 |  |
| POST9 | 88.33±55.89 | 149.10±63.81*^$^ | -68.79 (-113.40 to -24.19) | **0.0005** |  |
| ∆ change | -1.51±25.47 | 66.83±77.80 | -68.35 (-117.13 to -19.58) | **0.008** |  |
| MVPA (min/day) |  |  |  |  | **0.016** |
| PRE | 6.82±9.99 | 9.80±10.48 | -1.68 (-18.30 to 14.93) | 0.99 |  |
| POST3 | 6.71±9.79 | 16.90±9.77 | -8.26 (-26.81 to 10.28) | 0.77 |  |
| POST9 | 10.40±11.89 | 35.27±25.34*^$^ | -25.01 (-41.95 to -8.06) | **0.0009** |  |
| ∆ change | 3.59±17.13 | 25.80±10.48 | -23.63 (-42.91 to -4.34) | **0.01** |  |
| Total energy intake (Kcal/day) |  |  |  |  | 0.78 |
| PRE^a^ | 1162.3±273.8 | 1251.5±361.4 | 47.9 (-224.5 to 320.4) | 0.99 |  |
| POST3^b^ | 774.0±238.9 | 725.7±168.5 | -51.0 (-341.2 to 239.1) | 0.99 |  |
| POST9^b^ | 810.7±100.9 | 863.8±150.0 | -9.3 (-305.0 to 288.4) | 1.0 |  |
| ∆ change | -401.4±253.3 | -481.5±329.3 | 47.6 (-112.1 to 207.3) | 0.53 |  |
| Carbohydrate intake (g/day) |  |  |  |  | 0.71 |
| PRE^a^ | 139.11±17.82 | 149.62±37.83 | 3.33 (-32.40 to 39.08) | 0.99 |  |
| POST3^b^ | 91.66±33.83 | 85.03±28.52 | -10.07 (-46.34 to 36.18) | 0.95 |  |
| POST9^b^ | 101.53±17.81 | 111.12±39.06 | -0.79 (-39.86 to 38.26) | 1.0 |  |
| ∆ change | -40.94±18.46 | -47.39±33.48 | -2.49 (-36.21 to 31.22) | 0.87 |  |
| Protein intake (g/day) |  |  |  |  | 0.43 |
| PRE^a^ | 56.45±18.61 | 54.19±15.81 | -0.65 (-17.77 to 16.45) | 1.0 |  |
| POST3^b^ | 41.51±23.11 | 44.24±11.97 | 8.63 (-9.67 to 26.94) | 0.71 |  |
| POST9^b^ | 46.08±13.16 | 45.35±17.70 | 0.50 (-18.00 to 19.00) | 1.0 |  |
| ∆ change | -13.80±18.98 | -13.12±13.32 | 0.21 (-15.73 to 16.15) | 0.97 |  |
| Lipid intake (g/day) |  |  |  |  | 0.82 |
| PRE^a^ | 42.11±17.36 | 47.03±19.45 | 3.00 (-13.25 to 19.25) | 0.99 |  |
| POST3^b^ | 22.36±11.98 | 23.90±8.60 | -1.52 (-18.86 to 15.81) | 0.99 |  |
| POST9^b^ | 26.43±12.77 | 31.15±8.36 | 3.44 (-14.17 to 21.06) | 0.99 |  |
| ∆ change | -17.49±18.19 | -18.23±21.30 | 6.16 (-4.37 to 16.70) | 0.23 |  |
| Fiber intake (g/day) |  |  |  |  | 0.81 |
| PRE^a^ | 10.22±2.89 | 13.41±7.78 | 1.46 (-4.35 to 7.29) | 0.97 |  |
| POST3^b^ | 6.25±2.88 | 9.04±4.96 | 2.42 (-3.74 to 3.59) | 0.84 |  |
| POST9^b^ | 6.08±3.26 | 8.44±3.07 | 0.36 (-3.74 to 8.59) | 0.84 |  |
| ∆ change | -4.76±5.48 | -6.91±8.25 | 2.76 (-2.31 to 7.83) | 0.26 |  |

Data as mean±standard deviation, estimated mean of differences between RYGB + ET and RYGB (95% CI), level of significance (p=value; RYGB+ET x RYGB), and level of significance (interaction effect) calculated using a mixed model for repeated measures. Delta (∆) change = POST9 – PRE. Legend: BMI: Body Mass Index; VO_2peak_: peak oxygen consumption; 1-RM: 1 repetition maximum. Different superscript letters denote significantly different values for the main effect of time (p≤0.05). * means p≤0.05 for within-group comparisons (vs. PRE). ^$^ means p≤0.05 for within-group comparisons (vs. POST3).
