# Supplementary material for: Exercise reprograms the gut microbiota to enhance metabolic outcomes after bariatric surgery: a translational, cross-species study
Source: Int J Obes (Lond). 2026 Jun 4;50(10):2113–25. doi: 10.1038/s41366-026-02121-3 (PMC13722583; doi:10.1038/s41366-026-02121-3)
Supplement: Supplementary file 7 [file 41366_2026_2121_MOESM3_ESM.docx]

**Suppplementary Table 3.** Effects of exercise training on fecal short-chain fatty acids (SCFA) in women undergoing bariatric surgery

|  | **RYGB**  **(n= 16)** | **RYGB + ET**  **(n= 16)** | **Difference**  **(IC - 95%)** | **P-value** | **P-value**  **Group x Time** | **LEAN**  **(n=16)** |
| --- | --- | --- | --- | --- | --- | --- |
| Acetate (mmol/L) |  |  |  |  | 0.549 |  |
| PRE^a^ | 7.70±2.99 | 6.06±3.23 | -1.60 (-5.08 to 1.88) | 0.74 |  | 5.21±2.06 |
| POST3^b^ | 4.44±1.37 | 4.66±1.88 | 0.22 (-3.35 to 3.79) | 0.99 |  |  |
| POST9^ab^ | 5.55±1.50 | 5.14±1.88 | -0.40 (-3.06 to 2.24) | 0.99 |  |  |
| ∆ change | -1.99±4.56 | -0.53±4.14 | 0.46 (-1.91 to 2.84) | 0.67 |  |  |
| Propionate (mmol/L) |  |  |  |  | 0.862 |  |
| PRE | 2.91±1.51 | 2.35±1.88 | -0.61 (-2.64 to 1.41) | 0.94 |  | 1.60±0.71 |
| POST3 | 1.63±0.47 | 2.01±0.78 | 0.38 (-1.69 to 2.46) | 0.99 |  |  |
| POST9 | 2.42±1.30 | 2.47±1.23 | 0.05 (-1.49 to 1.60) | 0.99 |  |  |
| ∆ change | -0.51±2.76 | -0.09±2.99 | 0.34 (-1.30 to 1.99) | 0.65 |  |  |
| Butyrate (mmol/L) |  |  |  |  | 0.687 |  |
| PRE | 1.91±1.96 | 1.89±1.77 | -0.01 (-1.85 to 1.82) | 0.99 |  | 1.06±0.75 |
| POST3 | 1.06±0.38 | 1.20±0.60 | 0.14 (-1.74 to 2.02) | 0.99 |  |  |
| POST9 | 1.75±0.93 | 1.64±0.67 | -0.11 (-1.52 to 1.28) | 0.99 |  |  |
| ∆ change | -0.28±2.52 | -0.20±1.54 | -0.06 (-0.96 to 0.85) | 0.89 |  |  |

Data as mean±standard deviation, estimated mean of differences between RYGB + ET and RYGB (95% CI), level of significance (p=value; RYGB+ET x RYGB), and level of significance (interaction effect) calculated using a mixed model for repeated measures. Delta (∆) change = POST9 – PRE. Different superscript letters denote significantly different values for the main effect of time (p≤0.05).
