# Supplementary material for: Exercise reprograms the gut microbiota to enhance metabolic outcomes after bariatric surgery: a translational, cross-species study
Source: Int J Obes (Lond). 2026 Jun 4;50(10):2113–25. doi: 10.1038/s41366-026-02121-3 (PMC13722583; doi:10.1038/s41366-026-02121-3)
Supplement: Supplementary file 8 [file 41366_2026_2121_MOESM4_ESM.docx]

**Supplementary Table 4.** SCFA concentrations following human donor FMT (trained vs. non-trained bariatric vs. lean) in female SPF mice with diet-induced obesity

|  | **rRYGB**  **(n= 12)** | **rRYGB+ET**  **(n= 12)** | **rLEAN**  **(n=12)** | **p-value**  **Group** |
| --- | --- | --- | --- | --- |
| Acetate (mmol/L) | 3.70±1.18 | 4.29±0.94 | 4.07±2.44 | 0.69 |
| Butyrate (mmol/L) | 0.54±0.28 | 0.60±0.23 | 0.57±0.41 | 0.88 |
| Propionate (mmol/L) | 1.14±0.38 | 1.35±0.46 | 1.37±0.79 | 0.55 |

Data as mean±standard deviation, level of significance (group effect p-value) calculated using a mixed model for repeated measures.
