# Supplementary material for: Exercise reprograms the gut microbiota to enhance metabolic outcomes after bariatric surgery: a translational, cross-species study
Source: Int J Obes (Lond). 2026 Jun 4;50(10):2113–25. doi: 10.1038/s41366-026-02121-3 (PMC13722583; doi:10.1038/s41366-026-02121-3)
Supplement: Supplementary file 9 [file 41366_2026_2121_MOESM5_ESM.docx]

**SUPPLEMENTARY INFORMATION**

**MATERIALS AND METHODS**

**Human study**

***Physical capacity and physical activity levels***

Aerobic capacity was assessed using an incremental VO_2peak_ test (Centurion 200, Brazil) with minute-by-minute increments in speed and grade until volitional exhaustion. Gas exchange (VO_2_ and VCO_2_) was measured breath-by-breath and averaged every 30 s via indirect calorimetry (Cortex Metalyzer IIIB, Germany), while heart rate (HR) was monitored continuously using a 12-lead ECG. The test was deemed maximal if at least one criterion was met: VO_2_ plateau (e.g., <150 mL/min increase between stages), HR within 10 bpm of age-predicted maximum, or a respiratory exchange ratio >1.10 VO_2peak_ was defined as the average of the final 30 s of the test [1].

Following two familiarization sessions (separated by ≥72 h), lower- and upper-limb strength were assessed via 1RM leg-press and bench-press (Nakagym, Brazil), with coefficients of variation (CV) of 2.8% and 1.1%, respectively. Functional capacity was evaluated through the timed-stands test (max stands in 30s) and the timed-up-and-go test (time to stand, walk 3m, and return), with CVs of 2.1% and 3.5%, respectively. All functional assessments involved two attempts, and a single investigator conducted all tests to ensure consistency [2].

Physical activity and sedentary behavior were objectively measured via ActiGraph GT3X® accelerometers (ActiGraph, USA) for seven consecutive days during waking hours. Data were collected during non-training periods at PRE, POST3, and POST9. Participants wore the device on the right hip, maintained a daily wear-log, and were required to accumulate at least 10 h/day of valid recordings for at least 5 days. Data were processed in 60-s epochs using ActiLife 6 software. Non-wear time was defined as at least 60 min of zero counts (with a 2-min tolerance for counts <100). Following Freedson et al. [3] cut-points, activity was categorized as sedentary (<100 cpm), light-intensity (≥100 to <1952 cpm), or moderate-to-vigorous (≥1952 cpm). Sedentary and MVPA data are reported in minutes per day [3].

***Anthropometric measurements and body composition***

Patients were weighed on a digital scale, and their height was measured using a stadiometer in the standing position to calculate BMI. Waist and hip circumferences were measured with a measuring tape (Cescorf®). Body composition, including lean mass, fat mass, fat percentage, and bone mineral content, was assessed using a whole-body dual-source X-ray absorptiometry (DXA) scan (Lunar iDXA densitometer; GE Medical Systems, Madison, WI) and the CoreScan™ software.

***Biochemical parameters and inflammatory cytokines***

After a 12-h fast, blood was collected from the median or cephalic basilic vein for analysis of lipid markers (total cholesterol - TC; high-density lipoprotein - HDL; low-density lipoprotein - LDL; very low-density lipoprotein - VLDL and triglycerides - TG), glucose metabolism (glucose, C-peptide, insulin and glycated hemoglobin (HbA1C), and inflammatory markers C-reactive protein (CRP) and cytokines IL-1β, TNF-α, IL-6, and IL-10.

LDL and VLDL were calculated as previously described [4]. TG, TC, HDL, and CRP were assessed by colorimetric enzymatic methods (CELM, Sao Paulo, Brazil). Plasma glucose was assessed by a colorimetric enzymatic assay (Bioclin, Belo Horizonte, Brazil) and insulin was evaluated by a human-specific RIA method (Diagnostic Products Corp., Los Angeles, CA). C-peptide was assessed by chemiluminescent immunometric assays (Roche Diagnostics, Mannheim, Germany). Glycated hemoglobin (Hb_A1c_) was measured by high-performance liquid chromatography using ion exchange on a Biorad Variant II automated analyzer (Biorad, CA, USA). Cytokines were determined using a commercially available multiplex immunoassay kit (Lincoplex, Millipore, Missouri, USA) and quantified on a Luminex® analyzer (MiraBio, Alameda, CA) according to the manufacturer's instructions.

To assess insulin resistance, the Homeostatic Model Assessment for Insulin Resistance (HOMA-IR) index was calculated using fasting blood glucose and insulin levels. Moreover, an oral glucose tolerance test (OGTT) was performed. Blood was collected before and 30, 60, 90, and 120 minutes after the intake of 75 g of dextrose. The total area under the curve for insulin (AUC_insulin_) and glucose (AUC_glucose_) was calculated using the trapezoidal model.

***Food intake***

Dietary intake was assessed using three non-consecutive 24-hour food recalls (including one weekend day) and analyzed via Dietbox software. Standardized recipes were used to quantify complex preparations. Total energy intake (TEI; kcal) and macronutrient intake (g), including carbohydrates, lipids, protein, and fiber, were calculated.

***Fecal short-chain fatty acid (SCFA) analysis***

For SCFA analysis, 200-300 mg aliquots of the sample were collected, homogenized with 1 ml of 0.15 mM sulfuric acid, and centrifuged (9,000 g) at 4°C for 20 minutes. A 1 ml aliquot of the supernatant was collected, 1 ml of Milli-Q water was added, and filtered through a 0.45 µm nylon membrane (Millex^→^), after which they were injected into an Agilent gas chromatograph (model HP-6890), equipped with an Agilent selective mass detector (model HP-5975) using a DBWAX capillary column (60m× 0·25mm× 0·25 μm) under the following conditions: temperature of injector = 220°C, column = 35°C, 2°C/min, 38°C; 10°C/min, 75°C; 35°C/min, 120°C (1 min); 10°C/min, 170 °C (2 min); 40°C/min, 170 °C (2 min) and detector = 250 °C. Helium was used as a drag gas at a flow rate of 1 ml/min. Analytical curves were constructed using the stock solution of the acids of interest: acetic, propionic, and butyric acids. The samples were analysed in duplicate and data expressed as mmol/L of the final analytical extract [5].

**Animal study**

***Oral Glucose Tolerance Test (OGTT)***

The glucose tolerance test was performed following a 4-hour fast. Mice received a 50% sterile glucose solution (1g/kg of body weight) orally administered via gavage, and blood glucose from the tail was monitored at 0, 15, 30, 60 and 120 minutes. Blood glucose values were measured using a glucose monitor (AccuCheck Performa, Roche). Calculation of glucose response during the glucose tolerance test was estimated by total area under the glucose curve (AUC) using the trapezoidal method.

***Biochemical parameters and inflammatory cytokines***

Blood insulin concentrations were measured by radioimmunoassay and blood glucose was determined by a glucose monitor (Accu-Check Performa, Roche) to calculate the Homeostasis Model Assessment index (HOMA-IR) [6]. Plasma cytokines IL-1β, TNF-α, IL-6, and IL-10 were determined using a commercially available multiplex immunoassay kit (Lincoplex, Millipore, Missouri, USA) and quantified on a Luminex® analyzer (MiraBio, Alameda, CA) according to the manufacturer's instructions.

***Adipose tissue gene expression of inflammatory cytokines***

The expression of inflammatory cytokine genes was assessed using quantitative real-time polymerase chain reaction (qPCR). RNA was isolated from the adipose tissue using a commercially available kit (RNeasy Lipid Tissue Mini Kit; QIAGEN) according to the manufacturer´s protocol. mRNA quantification and purity assessment were performed using a spectrophotometer (NanoDrop 2000, Thermo Scientific). cDNA was obtained using High-Capacity cDNA Reverse Transcription Kit (Thermo Fisher Scientific, Massachusetts, EUA) according to the manufacturer´s protocol. cDNA amplification and detection were conducted in a real-time thermal cycler (Applied Biosystems 7500) using the SYBR Green dye (Applied Biosystems, catalog number 4367659). Reverse transcription quantitative polymerase chain reaction (RT-qPCR) was performed as previously described [7] using the following primer sets: IL-6, 5´-TACCACTTCACAAGTCGGAGGC-3´, 5´-AACTTTGGCATTGTGGAAGG-3´; IL10, 5´-CGGGAAGACAATAACTGCACCC-3´, 5´-CGGTTAGCAGTATGTTGTCCAGC-3´; TNF, 5´-GGTGCCTATGTCTCAGCCTCTT-3´, 5´-GCCATAGAACTGATGAGAGGGAG-3´; IL1ß 5´-GGACCTTCCAGGATGAGGACA-3´, 5´-GTTCATCTCGGAGCCTGTAGTG-3´. Cycle threshold (Ct) and ΔCt values were calculated in every sample for each gene of interest as follows: Ct^gene of interest^ − Ct^reference gene^, with ß-actin as the reference control gene. Relative changes in the expression level of the specific genes (Δ − ΔCq) were calculated by subtraction of the mean ΔCq of rLEAN to the ΔCq at POST (for all groups). Finally, fold change was determined as 2^−Δ−ΔCq^.

***Intestine morphological and histological analysis in mice***

These analyses were performed by a researcher blinded to the experimental groups. Jejunum and colon were arranged in Swiss-rolls and fixed in buffered formalin for 48 hours and stored in 70% alcohol at 4°C until processing, when they were embedded in paraffin for the assembly of histological blocks. Blocks were sectioned using a microtome to prepare histological slides and stained with hematoxylin-eosin. The depth of the crypts, height and width of the villi, and cellularity of each sample were evaluated. To standardize the analysis, 10 crypts and 10 villi from each sample were evaluated, and two pathologists performed the measurements in a double-blind evaluation system, where the pathologists were unaware of the distribution of the animals into groups. Morphological changes related to the inflammatory process, such as the presence of lymphoplasmacytic infiltrates, edema, and hyperplasia of Peyer's patches, were also evaluated. These changes were classified according to their intensity and extent and scored in both the jejunum and the colon samples. The histological score was defined as follows: 0 - no change; 1 - minimal; 2 - discreet; 3 - moderate; 4 - marked; 5 - severe.

***Fecal short-chain fatty acid (SCFA) analysis***

For SCFA analysis, 200-300 mg aliquots of the sample were collected, homogenized with 1 ml of 0.15 mM sulfuric acid, and centrifuged (9,000 g) at 4°C for 20 minutes. A 1 ml aliquot of the supernatant was collected, 1 ml of Milli-Q water was added, and filtered through a 0.45 µm nylon membrane (Millex^→^), after which they were injected into an Agilent gas chromatograph (model HP-6890), equipped with an Agilent selective mass detector (model HP-5975) using a DBWAX capillary column (60m× 0·25mm× 0·25 μm) under the following conditions: temperature of injector = 220°C, column = 35°C, 2°C/min, 38°C; 10°C/min, 75°C; 35°C/min, 120°C (1 min); 10°C/min, 170 °C (2 min); 40°C/min, 170 °C (2 min) and detector = 250 °C. Helium was used as a drag gas at a flow rate of 1 ml/min. Analytical curves were constructed using the stock solution of the acids of interest: acetic, propionic, and butyric acids.

**References**

1. Poole DC, Wilkerson DP, and Jones AM. Validity of criteria for establishing maximal O2 uptake during ramp exercise tests. Eur J Appl Physiol 102: 403-410, 2008.
2. Gil S, Kirwan JP, Murai IH, Dantas WS, Merege-Filho CAA, Ghosh S, Shinjo SK, Pereira RMR, Teodoro WR, Felau SM, Benatti FB, de Sá-Pinto AL, Lima F, de Cleva R, Santo MA, Gualano B, Roschel H. A randomized clinical trial on the effects of exercise on muscle remodelling following bariatric surgery. J Cachexia Sarcopenia Muscle. 2021;12(6):1440–1455. doi: 10.1002/jcsm.12783.
3. Freedson PS, Melanson E, Sirard J. Calibration of the Computer Science and Applications, Inc accelerometer. Med Sci Sports Exerc 1998;30:777–781.
4. Merege-Filho CAA, Gil SS, Kirwan JP, Murai IH, Dantas WS, Nucci MP, Pastorello B, Lima AP, Bazán PR, Pereira RMR, de Sá-Pinto AL, Lima FR, Brucki SMD, de Cleva R, Santo MA, Leite CDC, Otaduy MCG, Roschel H, Gualano B. Exercise modifies hypothalamic connectivity and brain functional networks in women after bariatric surgery: a randomized clinical trial. Int J Obes (Lond). 2023;47(3):165–174. doi: 10.1038/s41366-022-01261-5.
5. Dostal A, Baumgartner J, Riesen N, Chassard C, Smuts CM, Zimmermann MB, Lacroix C. Effects of iron supplementation on dominant bacterial groups in the gut, faecal SCFA and gut inflammation: a randomised, placebo-controlled intervention trial in South African children. Br J Nutr. 2014 Aug 28;112(4):547-56.
6. Matthews DR, et al. Homeostasis model assessment: insulin resistance and beta-cell function from fasting plasma glucose and insulin concentrations in man. Diabetologia. 1985;28:412–419. doi: 10.1007/BF00280883.
7. Baptista IL, Leal ML, Artioli GG, et al. Leucine attenuates skeletal muscle wasting via inhibition of ubiquitin ligases. Muscle Nerve. 2010;41(6):800–808. doi: 10.1002/mus.21612.

**SUPPLEMENTARY FIGURE LEGENDS**

**Supplementary Figure 1. Flowchart of patients**. RYGB+ET: Roux-en-Y Gastric Bypass plus Exercise Training group; RYGB: Roux-en-Y Gastric Bypass. ITT: intention-to-treat.

**Supplementary Figure 2**. **Effects of exercise training on gut microbiota α-diversity indexes in women undergoing bariatric surgery**. Average alpha diversity metrics (± 95% confidence intervals) in LEAN group and RYGB and RYGB+ET groups at PRE, POST3, and POST9 after surgery. * indicates within-group differences across time. No significant differences were found between groups (at fixed time points).

**Supplementary Figure 3**. **Effects of exercise training on gut microbiota β-diversity based on PCoA of Aitchison distances**. Colors and trajectories highlight time changes for each patient while groups are shape-coded. PERMANOVA results regarding RYGB and RYGB+ET groups are shown in the subtitle.

**Supplementary Figure 4. Venn diagram for the overall number of genera across FMT, PREFMT, and POST_FMT_ stool samples.**

**Supplementary Figure 5. Adipose inflammation markers following FMT from trained vs. non-trained donors**. Data are presented as mean±standard deviation of fold change. No differences were observed between groups.

**Supplementary Figure 6. Average α-diversity (±95% confidence intervals) in rLEAN, rRYGB, and rRYGB+ET groups before and after FMT**. * indicates significant within-group changes across time. No significant differences were found between groups (at fixed time points). Brown dots represent the corresponding alpha diversity computed on the FMT sample.

**Supplementary Figure 7. Beta diversity based on PCoA of Aitchison distances**. Colors and trajectories highlight time changes for each patient while groups are shape-coded. PERMANOVA results (for PREFMT and POSTFMT samples) are shown in the subtitle. The x-axis is truncated in the middle to improve the visualization of FMT samples, located in the left part of the plot.

**Supplementary Figure 8. Average centered-log-ratio values (CLR ± 95% confidence interval) for differentially abundant (DA) genera between and/or within GROUP at PRE_FMT_ and POST_FMT_**. * indicates within-group changes across time. No statistically significant differences were observed between groups. Brown dots represent the corresponding CLR values computed on the FMT sample. a. Genera where CLR values at the PRE_FMT_ were significantly lower than the corresponding value at POST_FMT_ in one or more groups. b. Genera where CLR values at the PRE_FMT_ were significantly higher than the corresponding value at POST_FMT_ in one or more groups.

**SUPPLEMENTARY TABLE LEGENDS**

**Supplementary Table 1.** Characteristics of medication among bariatric patients. Data expressed as absolute and relative frequency n (%). ACE: Angiotensin Converting Enzyme.

**Supplementary Table 2. Effects of exercise training on anthropometric measurements, body composition, and physical capacity and levels, and food intake in women undergoing bariatric surgery**. Data as mean±standard deviation, estimated mean of differences between RYGB + ET and RYGB (95% CI), level of significance (p=value; RYGB+ET x RYGB), and level of significance (interaction effect) calculated using a mixed model for repeated measures. Delta (∆) change = POST9 – PRE. Legend: BMI: Body Mass Index; VO_2peak_: peak oxygen consumption; 1-RM: 1 repetition maximum. Different superscript letters denote significantly different values for the main effect of time (p≤0.05). * means p≤0.05 for within-group comparisons (vs. PRE). ^$^ means p≤0.05 for within-group comparisons (vs. POST3).

**Supplementary Table 3. Effects of exercise training on fecal short-chain fatty acids (SCFA) in women undergoing bariatric surgery.** Data as mean±standard deviation, estimated mean of differences between RYGB+ET and RYGB (95% CI), level of significance (p=value; RYGB+ET x RYGB), and level of significance (interaction effect) calculated using a mixed model for repeated measures. Delta (∆) change = POST9 – PRE. Different superscript letters denote significantly different values for the main effect of time (p≤0.05).

**Supplementary Table 4. SCFA concentrations following human donor FMT (trained vs. non-trained bariatric vs. lean) in obese SPF mice**. Data as mean±standard deviation, level of significance (group effect p-value) calculated using a mixed model for repeated measures.
